# The initial pathways of broomcorn millet (*Panicum miliaceum*) into Europe based on AMS dates and stable carbon isotope data

**DOI:** 10.1038/s41598-026-70250-w

**Published:** 2026-09-04

**Authors:** Kyriaki Karanikola, Margaux L. C. Depaermentier, Tom Maltas, John Meadows, Dragana Filipović, Soultana-Maria Valamoti, Michael Kempf, Andrew Fairbairn, Michaela Vychronová, Goce Naumov, Sophia Koulidou, Clémence Pagnoux, Hanna Aleksandrova, Tzvetana Popova, Giedrė Motuzaite-Matuzeviciute

**Affiliations:** 1 Department of Archaeology, Faculty of History, Vilnius University, Vilnius, Lithuania; 2 Keble College, University of Oxford, Parks Road, Oxford, OX1 3PG UK; 3 School of Archaeology, University of Oxford, 1 South Parks Road, Oxford, OX1 3TG UK; 4 Leibniz-Zentrum Für Archäologie, Schloss Gottorf, Schlossinsel 1, 24837 Schleswig, Germany; 5 Leibniz Laboratory for AMS Dating and Stable Isotope Research, Christian-Albrechts-Universität, Max-Eyth-Strasse 11–13, 24118 Kiel, Germany; 6 ICArEHB, University of Algarve, Faro, Portugal; 7 DAE Research Group, Max Planck Institute of Geoanthropology, Jena, Germany; 8 LIRA Laboratory, Department of Archaeology, School of History and Archaeology and PlantCult Lab CIRI, Aristotle University of Thessaloniki, Thessaloniki, Greece; 9 Department of Geography, University of Cambridge, Cambridge, UK; 10 School of Social Science, The University of Queensland, Brisbane, Australia; 11 The Institute of Archaeology, Faculty of Arts, University of South Bohemia, České Budějovice, Czechia; 12 Laboratory of Archaeobotany and Palaeoecology, Faculty of Science, University of South Bohemia, České Budějovice, Czechia; 13 Center for Prehistoric Research-Skopje, Goce Delčev University, Shtip, North Macedonia; 14 Ephorate of Antiquities of Pieria, Greek Ministry of Culture, Katerini, Greece; 15 BioArch, CNRS–MNHN–Inrap, UMR 7209, CP 56, 57 Rue Cuvier, 75005 Paris, France; 16 National Archaeological Institute With Museum, Sofia, Bulgaria

**Keywords:** Broomcorn millet, AMS radiocarbon dating, Stable carbon isotopes, Crop introduction, Bronze age, Ecology, Ecology, Evolution, Solid Earth sciences

## Abstract

Research on broomcorn millet (*Panicum miliaceum* L.) has significantly improved our understanding of its introduction and dispersal across Europe during the second millennium BCE. However, its arrival into regions such as Southeastern Europe and Anatolia remains poorly understood. In the present study, we combine directly AMS-dated archaeobotanical millet grains with published stable carbon isotope data to investigate the chronology and dispersal routes of millet into Europe. The study integrates 70 radiocarbon dates obtained from charred millet grains, including 16 newly generated AMS dates, and re-evaluates previously published data using Bayesian chronological modeling. An extensive stable carbon isotopic dataset from human and animal remains is integrated in this review. The results confirm that the earliest secure evidence for millet in Europe currently derives from the western Pontic Steppe, while subsequent appearances are synchronously documented in the Carpathian Basin, Southeastern Europe, and the Caucasus. In contrast, archaeobotanical and isotopic evidence from Anatolia consistently supports a later introduction of millet in this region. These results reveal millet dispersal pathways into Europe and highlight the importance of direct radiocarbon dating and interdisciplinary approaches to study prehistoric crop dispersal and dietary practices.

**Supplementary Information:**

The online version contains supplementary material available at https://doi.org/10.1038/s41598-026-70250-w.

## Introduction

Broomcorn millet (*Panicum miliaceum*, hereafter “millet”) is widely recognized as one of the most important crops in the early stages of Eurasian food “globalization”. Together with foxtail millet (*Setaria italica*), it was domesticated in northern China ^1,2^ around 6000 BCE ^3^. After its domestication, millet spread across eastern and central Asia, becoming embedded in a wide range of subsistence systems across Eurasia ^4–7^.

The success of millet across Eurasia is closely linked to its particular agronomic properties. It is an annual crop tolerant of hot and dry conditions, poor soils, and limited water availability ^8^. Its short growing cycle, often only 60–70 days, made it attractive to mobile or semi-mobile groups and suitable for regions with short growing seasons or unpredictable rainfall ^8–10^. These characteristics have often been invoked to explain both its rapid spread and its association with risk-management strategies and agricultural flexibility ^6,11^. In Europe, it is associated with significant transformations in agriculture and diet, which are often framed within a broader “third food revolution” ^12^.

Several hypotheses have been proposed to retrace the westward dispersal routes of millet. Earlier studies emphasized a northern route along the Eurasian steppe corridor, largely based on indirect evidence ^11,13^. More recent data from the southern Caspian region and the Caucasus suggest the possibility of additional southern pathways in western Asia during the second millennium BCE ^14,15^.

For this period, archaeobotanical evidence from Anatolia remains limited and chronologically uncertain, as directly radiocarbon-dated millet grains are extremely scarce ^9,15,16^. Nevertheless, the small number of finds has been used to support the possibility of a southern route through which millet may have reached the Aegean, likely following its initial spread into Europe from the north ^17^. Millet appears relatively late and sporadically in Anatolia and may have been introduced from the north via the Caucasus rather than through western or eastern routes ^16^. This interpretation is consistent with its absence from Hittite textual records and major storage contexts, as well as from much of the archaeobotanical record of the second millennium BCE in Anatolia ^18^.

For Europe, the chronological framework primarily relies on directly dated macroremains from the western Pontic Steppe (northern Black Sea region), the Carpathian Basin, and Central Europe ^19–21^. Significantly fewer data have been published from Southeastern Europe, hindering our understanding of the initial millet pathways into these parts of Europe. The earliest securely dated millet grains in Europe come from present-day Ukraine and date to the sixteenth century BCE ^20^. Within roughly a century, millet spread into the Carpathian Basin, Central Europe, and Southeastern Europe ^11,20,22,23^. It reached large parts of northern and western Europe by the twelfth century BCE ^24^ and only the northernmost margins by the tenth century BCE ^25,26^.

Archaeobotanical evidence documents the presence of the crop, whereas stable isotope analyses of human and animal skeletal tissues reflect its actual consumption—provided that millet was the only possible source of C_4_ food and that it represented a substantial part of the diet ^27^. Stable isotope data from human bone and dentine collagen have thus recorded the earliest consumption of millet as a staple crop in Eastern and Central Europe in the middle of the fifteenth century BCE ^28–31^. This is around one century after the earliest evidence of directly dated millet grains in the Pontic Region but simultaneous with the archaeobotanically attested spread across Central Europe ^20^. Recent paleoproteomic analyses of human dental calculus, which can identify ancient proteins from specific foods preserved on human teeth, have further suggested the possibility of broomcorn millet consumption in parts of the Pontic–Caspian region considerably earlier than hitherto expected, i.e., in the 4th–3rd millennia BCE ^32^. However, it does not yet constitute direct evidence for the widespread cultivation and integration of millet into agricultural systems.

Despite these advances, the pathways through which millet entered Europe remain unclear, as significant geographical and chronological gaps persist. A key unresolved question is whether millet entered Europe primarily via the Pontic Steppe or through alternative southern pathways involving the Caucasus and Anatolia and what role Anatolia may have played in this process.

**Fig. 1:**
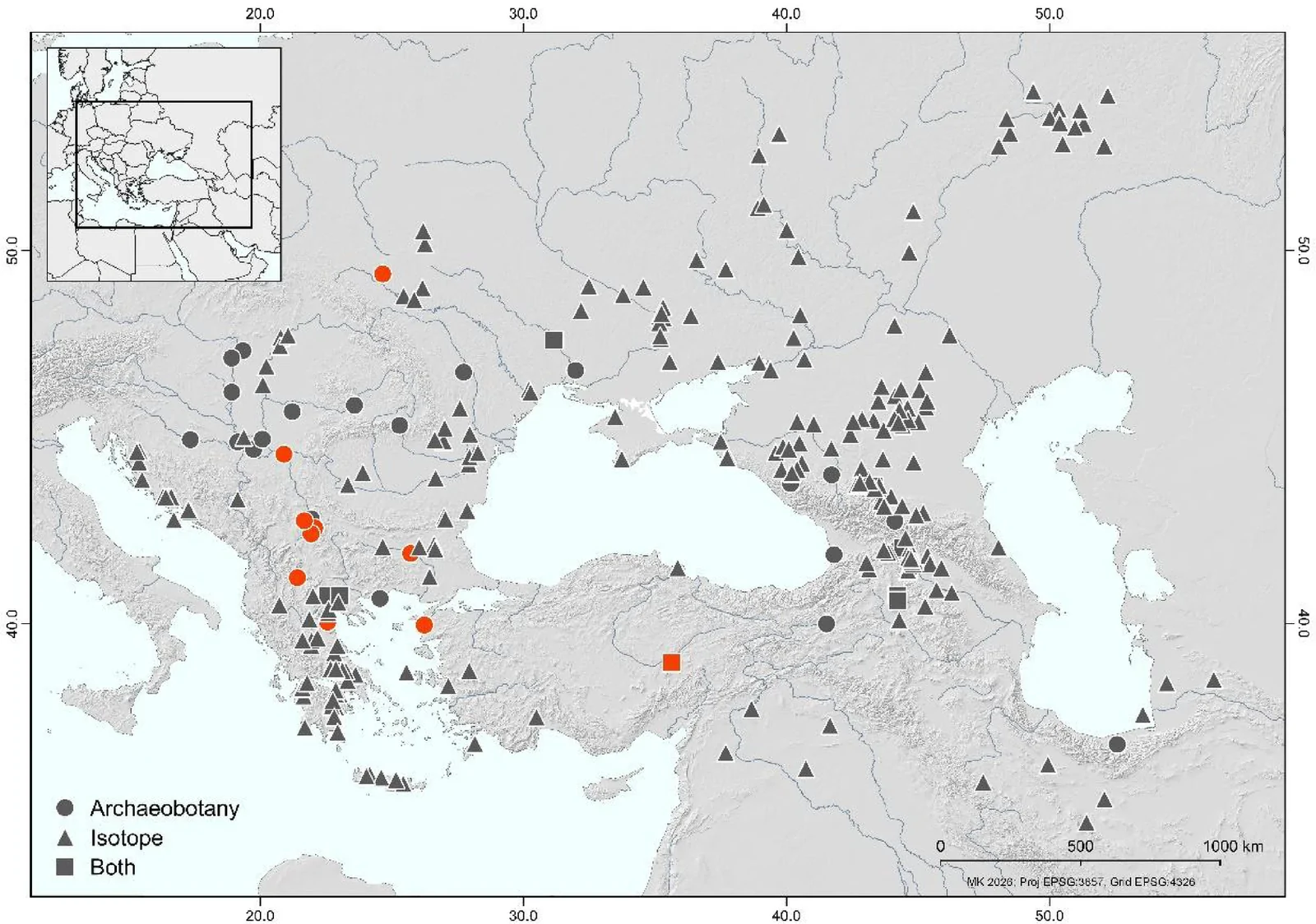
Geographic distribution of archaeobotanical and stable carbon isotope data included in this study. The map shows sites across the study area. Symbol shape indicates dataset type: circles represent archaeobotanical evidence of directly radiocarbon-dated broomcorn millet grains, triangles indicate stable isotope data, and squares mark sites where both datasets are available. Symbol color distinguishes data origin: red symbols denote newly generated radiocarbon dates, while grey symbols represent previously published data. Full references for the archaeobotanical and isotopic datasets are provided in Data S1 (see also ^96^) and Data S2 (see also ^176^), respectively.

To address these gaps, we present new radiocarbon dates from regions that have so far been under-represented, together with a synthetic reassessment of directly dated archaeobotanical millet remains, integrated with published stable carbon isotope data from human and animal skeletal tissues. The study area spans from the Adriatic to the eastern coast of the Caspian Sea and from Ukraine to Crete (Fig. 1). The second millennium BCE (ca. 2200/2000–1200/1000 BCE) forms the primary chronological focus, as this interval encompasses the initial appearance and spread of millet into Europe.

This unprecedented large-scale, long-time, and multi-proxy review is made possible by the plethora of local to regional datasets that are now available in open access and that can be combined to get insights into the bigger picture. At the continental scale, we therefore investigate the pathway of millet distribution, while at the regional scale, we determine the link or discrepancy between presence and human or animal consumption of millet by comparing archaeobotanical and isotopic evidence.

## Results

**Fig. 2:**
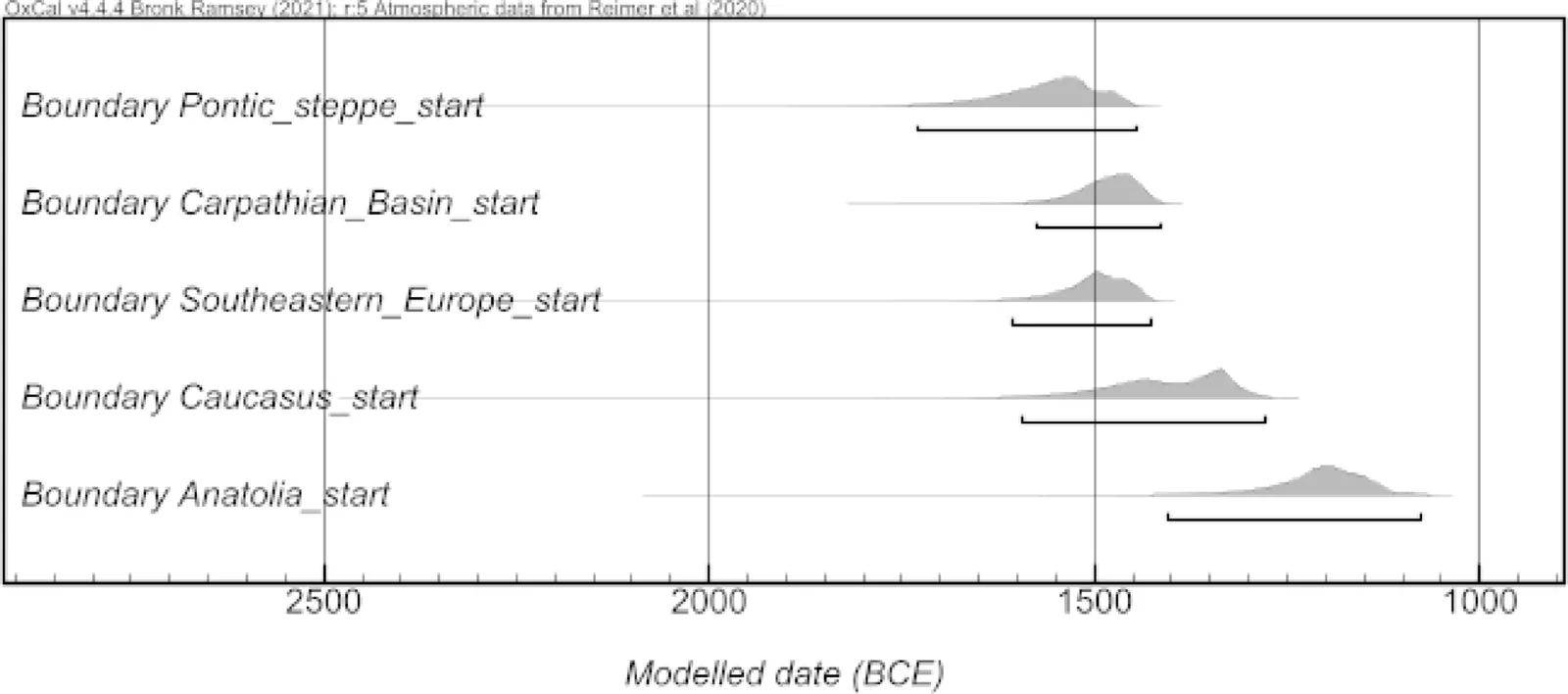
Posterior probability distributions of modeled start Boundaries representing the earliest probable appearance of directly dated broomcorn millet across the studied regions, produced using OxCal v4.4.4 ^97,98^ and the IntCal20 calibration curve ^99^. Only the start Boundaries from the Bayesian models are shown.

Bayesian modeling of directly dated millet grains provides posterior estimates for the earliest probable appearance of millet within the investigated regions. The model output is illustrated in Fig. 2. The earliest modeled occurrence derives from sites located in present-day Ukraine in the northern Black Sea region. The posterior start Boundary of the Pontic Steppe falls between 1614–1494 cal BCE (68.3%) and 1724–1446 cal BCE (95.4%). Southeastern Europe, the Carpathian Basin, and the Caucasus show closely overlapping chronologies.

The modeled onset in Southeastern Europe falls between 1526–1446 cal BCE (68.3%) and 1608–1426 cal BCE (95.4%). In the Carpathian Basin, it falls between 1560–1450 cal BCE (68.3%) and 1688–1424 cal BCE (95.4%), while in the Caucasus, it falls between 1466–1316 cal BCE (68.3%) and 1593–1276 cal BCE (95.4%).

The modeled onset in Anatolia falls between 1252–1137 cal BCE (68.3%) and 1402–1068 cal BCE (95.4%). Given the small number of directly dated millet grains in Anatolia, these estimates should be interpreted with caution.

In parallel, the isotopic data show a wide range in every region, as depicted in Supplementary Fig. S1 for collagen data and in Supplementary Fig. S2 for apatite data. The collagen *δ*^13^C values (n = 3274) overall range from  − 25.9 to − 7.6‰ across time and space (1st Qu.: − 20.0‰, median: − 19.4‰, 3rd Qu.: − 18.6‰), with the Pontic Steppe (n = 1012, min.: − 24.4‰, max.: − 9.2‰, 1st Qu.: − 20.2‰, median: − 19.4‰, 3rd Qu.: − 18.5‰) and Southeastern Europe (n = 1353, min: − 25.9‰, max: − 7.6‰, 1st Qu.: − 20.0‰, median: − 19.5‰, 3rd Qu.: − 18.8‰) showing the widest ranges for collagen *δ*^13^C, and Anatolia (n = 256, min: − 23.0‰, max: − 16.2‰, 1st Qu.: − 20.1‰, median: − 19.8‰, 3rd Qu.: − 19.4‰), showing the narrowest range among the main investigated regions (Supplementary Fig. S1).

As for bone and enamel apatite, the full dataset (n = 787) shows *δ*^13^C values ranging from − 15.1‰ to − 5.1‰ (1st Qu.: − 12.0‰, median: − 11.4‰, 3rd Qu.: − 10.8‰) (Supplementary Fig. S2). Here, the Caucasus dataset (n = 644) shows the greatest range (min.: − 14.2‰, max.: − 15.1‰, 1st Qu.: − 11.7‰, median: − 11.3‰, 3rd Qu.: − 10.7‰), while only Southeastern Europe provides another large dataset (n = 188).

## Discussion

### Millet introduction into Europe

Based on Bayesian chronological modeling (Fig. 2), the earliest directly dated evidence for millet in Europe is concentrated in the Pontic Steppe, in present-day Ukraine (1724–1446 cal BCE, 95.4%), refining the currently available chronological framework ^20^. Directly dated millet grains from the Carpathian Basin, Southeastern Europe, and the Caucasus follow within broadly overlapping mid-second millennium BCE ranges (Fig. 2; see also ^15,20,22,23,33^). In contrast, directly dated evidence from Anatolia is consistently later than in the other regions (Table 1; see also ^15^).

**Table 1:** Newly obtained direct AMS radiocarbon dates on charred broomcorn millet (*Panicum miliaceum*) grains. Radiocarbon ages are reported in years BP (± 1σ). Calibrated age ranges are given at the 95.4% probability level. The final column indicates whether each determination was included in the Bayesian chronological model. For the full list of the AMS dates, see Data S1 (see also ^96^).

Country	Site	Lab Code	14C age (BP ± 1σ)	Calibrated age (calBCE/CE, 95.4%)	Included model (yes/no)
Bulgaria	Nova Nadejda	FTMC-SW48- 2	2804 ± 32	1047–841 BCE	yes
Greece	Rema Xydias	FTMC-OG79-1	3046 ± 31	1403–1220 BCE	yes
Greece	Rema Xydias	FTMC-OG79-2	3047 ± 34	1410–1216 BCE	yes
North Macedonia	Vrbjanska Cuka	FTMC-SW48-3	1757 ± 30	235–384 CE	no
North Macedonia	Vrbjanska Cuka	FTMC-SW48-4	1837 ± 30	125–315 CE	no
Serbia	Piljakovac-Kržince	KIA-56389	3060 ± 30	1412–1227 BCE	yes
Serbia	Ranutovac	KIA-56387	2560 ± 29	805–567 BCE	yes
Serbia	Ranutovac	KIA-56388	2630 ± 45	905–596 BCE	yes
Serbia	Skorenovac	KIA-56391	1965 ± 90	196 BCE-310 CE	no
Serbia	Skorenovac	KIA-56390	1900 ± 45	25–236 CE	no
Serbia	Svinjarička Čuka	KIA-56392	3166 ± 28	1503–1397 BCE	yes
Serbia	Svinjarička Čuka	KIA-56393	3190 ± 40	1533–1328 BCE	yes
Ukraine	Zalukva	FTMC-JA88-2	2185 ± 32	371–123 BCE	no
Türkiye	Troy	FTMC-US70-1	2885 ± 33	1201–935 BCE	yes
Türkiye	Troy	FTMC-US70-2	2959 ± 31	1267–1052 BCE	yes
Türkiye	Kültepe	FTMC-LI18-1	2222 ± 35	386–197 BCE	no

**Fig. 3:**
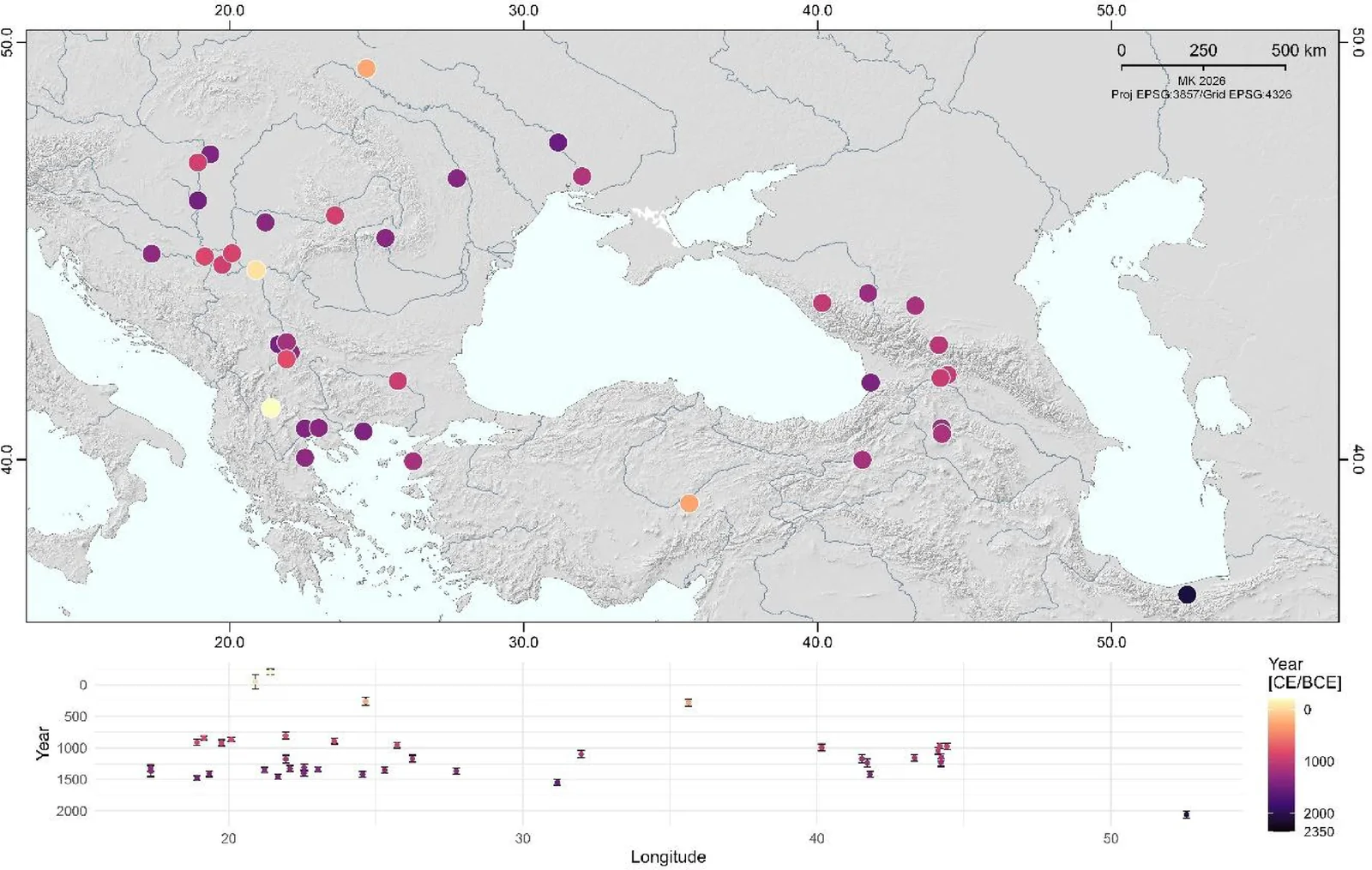
Distribution of archaeological sites with directly radiocarbon-dated broomcorn millet remains, showing only the earliest available radiocarbon determination from each site. Colors correspond to the mean calibrated age (BCE/CE) of the earliest dated millet grain; mean values are presented for consistency with the visualization approach applied to the isotopic dataset. The lower panel illustrates the distribution of these earliest site dates across longitude, with the color showing the mean date and error bars showing the dating range (SD).

The Caucasus region has previously been proposed as a corridor linking Southwest Asia ^14^ and Eastern Europe ^15^, through which millet dispersed into Eastern Europe. However, directly dated millet grains from the Caucasus are broadly contemporaneous with those from the Carpathian Basin and sites in Southeastern Europe and appear later than the earliest currently documented occurrences in the Pontic Steppe (Fig. 3). A concentration of millet grains reported from Tabakoni, in present-day Georgia ^34^ was associated with a wood fragment radiocarbon-dated to 1877–1686 cal BCE (95.4%). These indirectly dated grains may point to an earlier presence of millet in the Caucasus, but their chronology cannot be considered equivalent to that of directly AMS-dated single grains (see Methods).

**Fig. 4:**
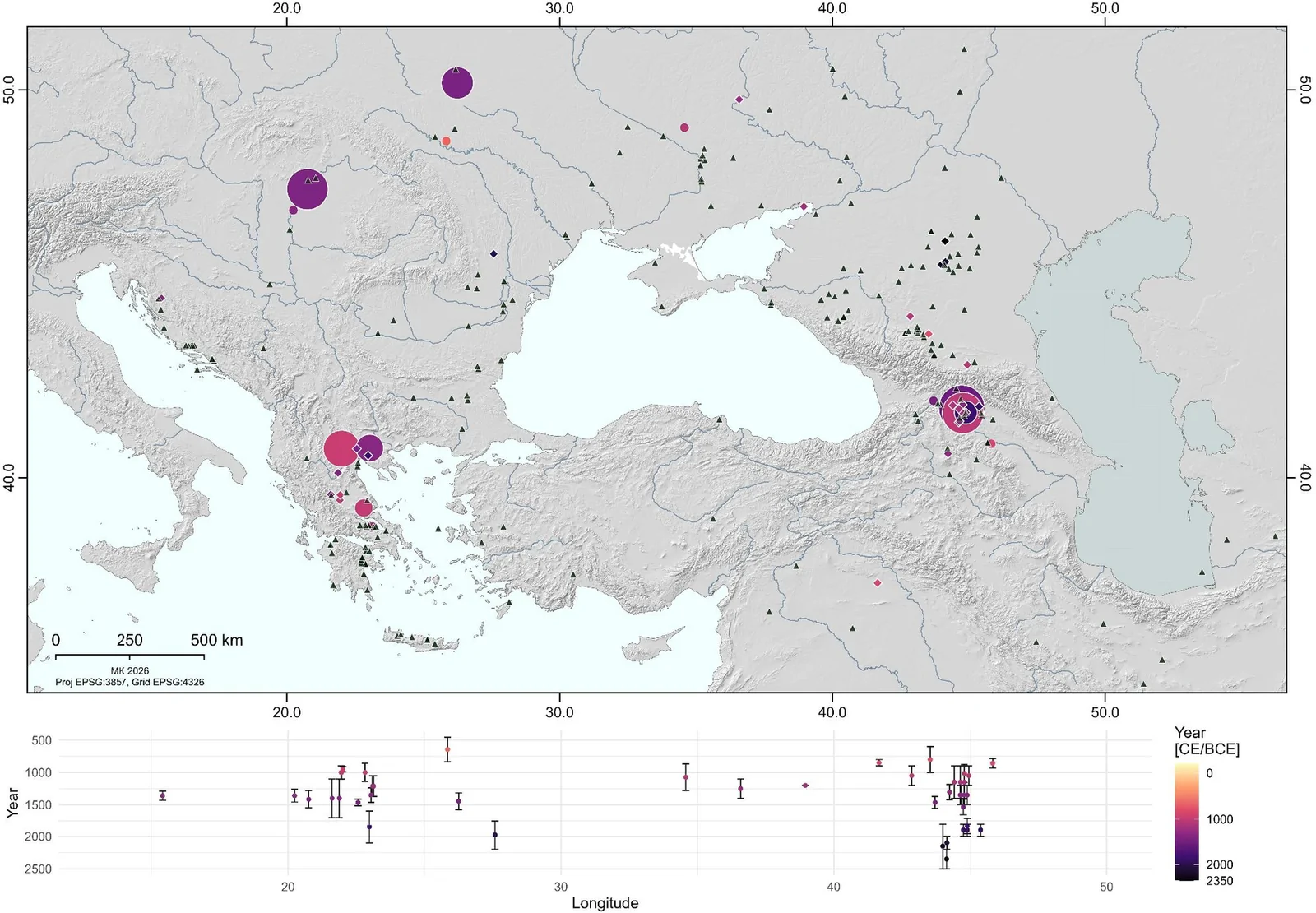
Distribution of archaeological sites with carbon isotopic evidence for C_3_ diet versus possible C_4_ diet. Colored signatures reflect the mean date (cal) BCE associated with carbon isotopic evidence for C_4_ consumption. The shape of the dots represents the dating method: circles for radiocarbon-dated samples and diamonds for archaeologically dated samples. The size of the circles reflects the proportion of human or animal individuals showing elevated *δ*^13^C signals associated with C_4_ diet at the site—yet the smallest size shows sites where no percentage could be assessed due to the small sample size. Grey triangles represent evidence for C_3_ diet only. The lower panel illustrates the chronological distribution of the isotopic evidence for C_4_ diet across longitude, with the color showing the mean date and error bars showing the dating range.

In terms of stable carbon isotopic evidence (Data S2, see also ^176^), an elevated apatite *δ*^13^C value (− 8.2‰) from a bovid at Goytepe (Azerbaijan) is archaeologically dated to around 5500 BCE ^35^ and therefore predates any expectation for millet presence (Supplementary Fig. S3). Animal collagen samples from Georgia show *δ*^13^C values as high as expected for millet consumption and date to around 3000 cal BCE, yet they likely reflect the impact of locally available wild C_4_ intake instead of millet consumption ^36^. The elevated collagen *δ*^13^C values from the Caucasus that may be more securely associated with millet consumption roughly belong to the period around 2000–1750 BCE (Fig. 4), e.g., from archaeologically dated samples from Atskuri (up to − 16.6‰, 2000–1800 BCE), Tsitsamuri (up to − 15.2‰, 2000–1800 BCE), and Gantiadi (up to − 15.5‰, 2200–1700 BCE), and from a radiocarbon-dated sample from Karataki (− 16.1‰, 1921–1748 cal BCE) ^15^. This predates the archaeobotanical evidence from the Caucasus by at least two centuries and could be contemporaneous to the earliest macro-remains of the Pontic Steppe (Figs. 2 and 3). A potential explanation for this result is that millet was primarily consumed by mobile communities for which settlement and hence archaeobotanical remains are rarely recorded today ^4,19^, implying that millet consumption would be mainly visible from the skeletal remains.

In turn, elevated collagen *δ*^13^C values as old as 4500–3500/3000 BCE are documented throughout the Pontic Steppe ^37–39^ (Supplementary Fig. S3). However, these are not only related to archaeologically dated samples—except for the sample from Peshtchanka (Ukraine) ^38^—but have also been previously interpreted as reflecting the impact of locally available C_4_ plants, of fish consumption, or of aridity instead of any evidence for millet consumption (e.g., ^37,38,40,41^). Moreover, such evidence has been considered too early for millet evidence due to current models ^7,20,42^. However, recent paleoproteomic research has presented direct evidence for millet consumption close to the north-eastern Black Sea coast as old as 3341–2203 cal BCE and as old as 4899–3818 cal BCE towards the north-eastern edge of our research area ^32^. This evidence demonstrates millet consumption at the individual level but does not by itself establish local cultivation or the importance of millet within the wider population. Hence, interpreting elevated *δ*^13^C values as millet consumption for such early material cannot be considered unlikely anymore. More studies investigating links between diverse types of subsistence practices and millet consumption (e.g., ^19^) and integrating information on mobility patterns are needed to understand this discrepancy between proxies.

Further archaeologically dated human and animal samples for the period around 2500–2200 BCE Peschany ^41^, 2500–1800 BCE (Sharakhalsun; ^43^ ) and 2200–2000 BCE (Ostrovnoy; ^44^ ) in the eastern margin and around 2200–1600 BCE in the western margin of the Pontic Steppe (Cârlomănești; ^45^) (Fig. 4) show elevated *δ*^13^C values that could possibly reveal millet consumption, although the particularly dry conditions and the local presence of wild C_4_ plants could also have triggered such *δ*^13^C values. This potential evidence is at least two centuries before the archaeobotanical evidence from the northern Black Sea region (Fig. 3) and up to one millennium after the evidence from protein analyses ^32^. Therefore, millet consumption cannot be excluded in those early contexts, but it is not securely proven by means of stable carbon isotopes. The radiocarbon-dated human sample from Igren VIII (Ukraine) that would best fit with the archaeobotanical model is dated to 1413–1278 cal BCE ^46^. Yet it has a collagen *δ*^13^C value of − 17.1‰, i.e. too close to the local threshold for C_4_ intake identification ^47^, and a too-elevated *δ*^15^N value (12.2‰) to be securely associated with millet consumption.

Overall, the combined paleoproteomic ^32^, archaeobotanical, and isotopic evidence indicates that the earliest secure presence of millet in Europe is best supported in the Pontic Steppe. This suggests that millet reached Europe via the Pontic Steppe to the north of the Caspian Sea, followed by a relatively rapid expansion across neighboring regions (i.e., the Caucasus, Carpathian Basin, and Southeastern Europe) during the mid-second millennium BCE, rather than by following a simple or sequential dispersal along a single clearly defined pathway.

### Millet dispersal into Central and Southeastern Europe

Beyond the Pontic Steppe, millet appears in Central and Southeastern Europe within broadly overlapping mid-second millennium BCE chronological ranges, with the earliest directly dated evidence in the Carpathian Basin falling between 1688 and 1424 cal BCE (95.4%) and in Southeastern Europe between 1608 and 1426 cal BCE (95.4%) (Figs. 2 and 3). Stable carbon isotopic evidence for millet consumption in the Carpathian Basin is found in human collagen samples from Tiszafüred-Majoroshalom (Hungary), radiocarbon-dated to 1510–1320 cal BCE at the earliest (Fig. 4), whereas remains (starch) are found in human dental calculus dated to 1740 − 1540 cal BCE ^28^. These data are consistent with the earliest archaeobotanical evidence based on directly dated macroremains (e.g., 1688–1424 cal BCE), although further isotopic research is needed to confirm this pattern.

In Southeastern Europe, the contemporaneity between archaeobotanical and isotopic records is less straightforward to evaluate, as both datasets spatially hardly overlap (Fig. 1). Elevated collagen *δ*^13^C values (between − 15.6 and − 17.3‰ in Bulgaria and Romania, or at − 16.6‰ in Greece) are found in several contexts dated between 2900 and 2100 BCE ^38,48,49^ (Supplementary Fig. S3) and were identified in remains as early as c. 4545–4179 cal BCE (Gumelnița, Romania) ^50^. Yet they are too close to the local threshold ^47^ to be securely associated with millet consumption and might be caused by wild C_4_ plants available from these regions ^50^, while millet grains attested from archaeobotanical remains of the same period in Romania are not radiocarbon-dated ^51^. The early faunal collagen sample from Thebes (southern Greece), showing a *δ*^13^C value of − 15.3‰, is furthermore associated with seaweed consumption ^52^.

The faunal collagen sample from Toumpa Thessaloniki (*δ*^13^C up to − 12.6‰), archaeologically dated to 2100–1600 BCE (Fig. 4), may be associated with millet consumption ^48,53^, yet local C_4_ vegetation or the consumption of marine food might have caused this signal as well ^48,53,54^. Further early isotopic evidence for possible millet consumption in Greece is archaeologically dated to 1700–1100 BCE and restricted to the northern part, with Rymnio in Greek Macedonia ^55^ and Treis Elies in Pieria ^56^. The southernmost evidence comes from one single individual from Kolaka, Central Greece, archaeologically dated to 1325–1100 BCE ^57,58^. Without a radiocarbon date, it is, however, challenging to compare those results with the archaeobotanical records.

The only radiocarbon-dated evidence for elevated *δ*^13^C values in Greece comes from Assiros (*δ*^13^C value of − 16.1‰) and is dated around 1430–1272 cal BCE ^59^. This value remains close to the local threshold defined at c. − 16.0 ± 0.8‰ ^47^, maybe representing a small dietary intake, while more elevated collagen *δ*^13^C values (between − 15.4 and − 11.9‰) are attested at the site and overall radiocarbon-dated between 1394 and 1012 cal BCE ^59^. At Assiros, directly radiocarbon-dated millet grains fall within a chronological range comparable to the isotopic evidence ^20^, while the broader archaeobotanical record indicates a marked increase in millet presence after the middle of the second millennium BCE, with particularly high proportions recorded in the settlement phase dated to 1300–1100 BCE ^59–61^. Both archaeobotanical and isotopic data thus suggest a progressive intensification in millet consumption.

According to the Bayesian model, millet appeared in the Carpathian Basin and Southeastern Europe in broadly contemporaneous chronological frameworks during the mid-second millennium BCE. Overall, the available evidence indicates a closer alignment between archaeobotanical and isotopic data in the Carpathian Basin, while in Southeastern Europe, the interpretation of isotopic signals remains more complex due to environmental influences and a lack of spatially overlapping datasets. In contrast to the pattern observed in the Pontic Steppe, the synchronous evidence for millet consumption from archaeobotany and stable carbon isotopes in Central and Southeastern Europe suggests that millet was introduced into established sedentary agricultural systems when reaching these regions.

### Evaluating the Anatolian route

By contrast, both archaeobotanical and stable carbon isotope evidence indicate a delayed arrival of millet in Anatolia compared with the other regions, with the modeled onset falling between 1402 and 1068 cal BCE (95.4%) (Fig. 2). Millet grains from archaeological contexts predating the twelfth century BCE have been recorded from seven sites across Anatolia ^17^. However, their very small sample size (< 15 grains) and the presence of later occupation layers, with some containing much larger quantities of millet, suggest that these early finds represent intrusions (e.g., ^11,21^). This interpretation is further supported by archaeobotanical evidence from extensively investigated central Anatolian sites such as Kaman-Kalehöyük, Büklükale, and Kültepe, where millet remains are extremely rare in Bronze Age contexts despite the recovery of large archaeobotanical assemblages, while increased ubiquity and frequency are only observed during the first millennium BCE ^62–64^.

Indeed, directly radiocarbon-dated millet grains in this study from western and central Türkiye consistently yielded later dates than expected from the archaeological context (Table 1) and are overall approximately 200–300 years later than the earliest evidence from the Pontic Steppe and other parts of Europe (Figs. 2, and 3).

As for the stable isotopic records, only seven out of the 256 samples from Anatolia show collagen *δ*^13^C values above − 18.0‰ (Data S2, Supplementary Fig. S1). Those from the Bronze Age sites at Kültepe (− 17.9 and − 17.6‰), Kaymakçı (− 17.6 and − 16.9‰), and Bakla Tepe (− 17.6‰) are too close to the theoretical threshold—especially if considering the potentially higher threshold at around − 17 or − 16‰ derived from recent interpolations ^47^—and are hence still considered as C_3_ diet ^65,66^ (Supplementary Fig. S3). In contrast, the most elevated collagen *δ*^13^C values are found in single animals, i.e. a roe deer from Aşıklı Höyük (− 12.7‰; c. 8350 − 7300 BCE) ^67^, a cow from Barcın Höyük (− 15.8‰; c. 6600–6000 cal BCE) ^68^, and a cow from Bademağacı (− 16.2‰, c. 2600–2300 BCE) ^69^. These are mostly outlier animals among other samples that exhibit typically C_3_ diets and have been interpreted as having access to wild C_4_ plants ^68,69^. The C_4_ isotopic signal identified in Neolithic humans from Çatalhöyük was also interpreted as the indirect impact of seasonally available wild C_4_ plants via the consumption of meat ^70^ instead of millet intake. Moreover, these samples are often only archaeologically dated. And since the oldest human with elevated *δ*^13^C values (− 15.71‰) that was found at the Neolithic site at Nevalı Çori (c. 7500–6300 BCE) turned out to be post-Neolithic based on the stratigraphy ^71^, non-radiocarbon-dated records need to be considered with caution in this region as well.

To the best of our knowledge, all other isotopic studies based on Neolithic, Chalcolithic or Bronze Age material overall show a prevailing pure C_3_ diet throughout the region (e.g., ^66,69,72–75^). Our isotopic review, therefore, suggests a lack of evidence for any millet consumption during the expected period of millet spread. Despite the early presence of millet in West Asia, with radiocarbon-dated macroremains dating to around 2000 cal BCE at Ghal e-Ben on the southern coast of the Caspian Sea in northern Iran ^14^, its westward spread does not currently appear to have continued through Anatolia.

Consequently, although a southern route via the southern Caspian cannot be entirely excluded, as potentially suggested by early yet ambiguous elevated *δ*^13^C values along the southern Caspian littoral ^76^ and in northern Iran and northern Syria ^77,78^ (Supplementary Fig. S3), the currently available archaeobotanical and isotopic evidence does not provide actual support for an Anatolian pathway during the initial spread of millet into Europe. Further research, particularly through securely dated archaeobotanical and isotopic datasets, is required to better evaluate potential southern dispersal routes.

### Patterns of millet adoption

Beyond its initial dispersal, archaeobotanical evidence indicates that millet played a variable and context-dependent role within later prehistoric cropping systems, with signs of increasing importance from the mid-second millennium BCE onwards (e.g. ^20,23^).

In the Caucasus, directly radiocarbon-dated millet remains derive primarily from contexts with small quantities, reinforcing the picture of an initially limited presence ^15^.

In Southeastern Europe and the Carpathian Basin, in some cases the quantity and frequency of millet remain increase in later phases at sites with long occupational sequences, such as Assiros (Greece), Sovjan (Albania), and Feudvar (Serbia) ^59–61,79,80^. In addition, large quantities of millet are recorded at the end of the second millennium BCE at sites such as Kalnik-Igrišče (Croatia) ^22,81^. Comparable trends have been reported in sites in Bulgaria, where millet is more frequently attested in later contexts, particularly towards the end of the second and the beginning of the first millennium BCE (e.g., ^82^).

Overall, archaeobotanical evidence suggests a gradual and regionally differentiated intensification of millet use, rather than a synchronous or uniform process of adoption. However, this pattern is not uniform across sites and may partly reflect differences in research intensity and preservation conditions.

**Fig. 5:**
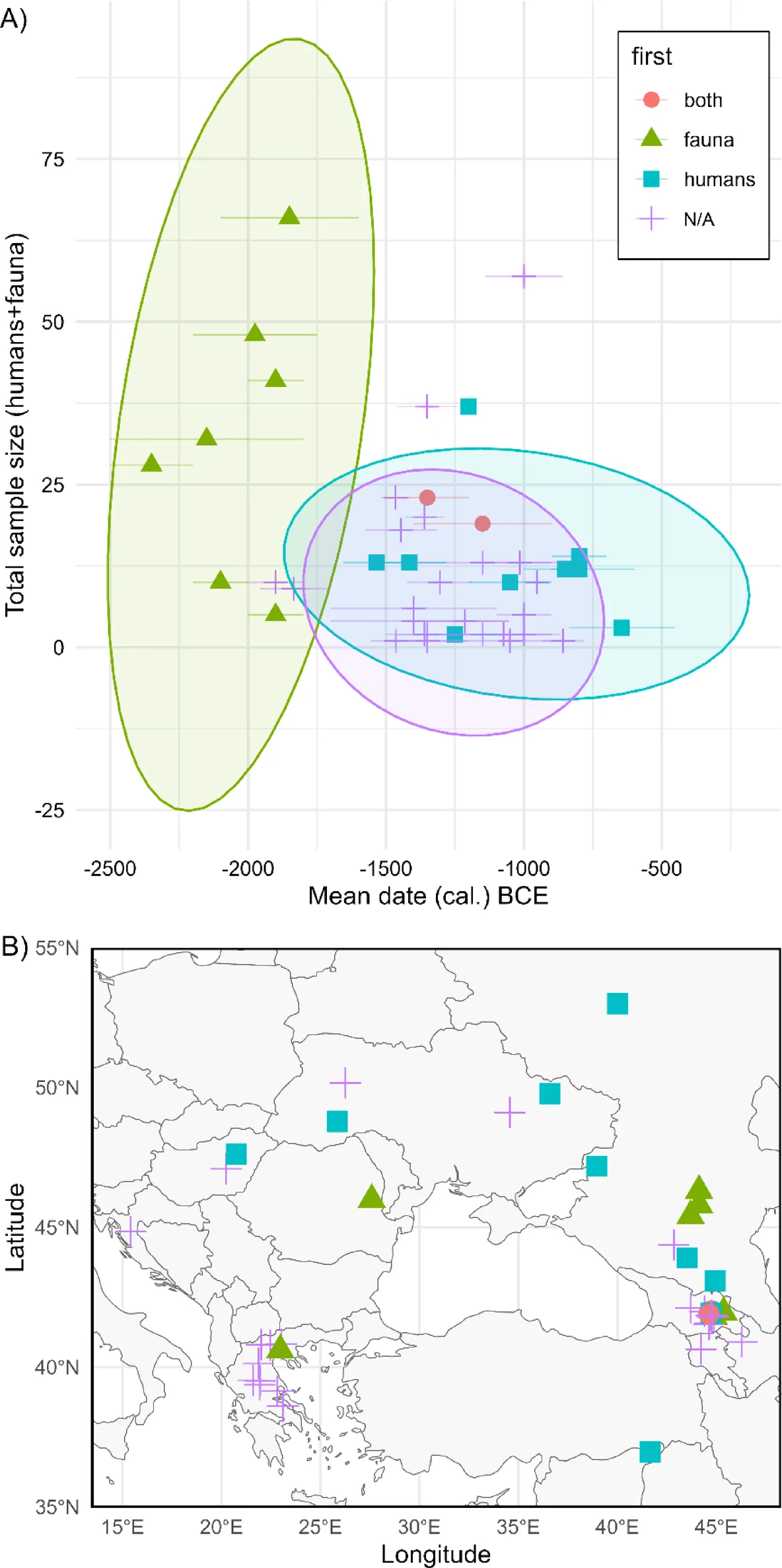
Spatio-temporal framework of sites at which a C_4_ diet was identified either first in humans or first in animals, or in both at the same time. A) Chronological evolution compared with the total sample size (including humans and animals). Ellipses depict data dispersion at the probability level of 95% of each category. B) Spatial distribution. The legend applies for both A) and B). Sites marked as “N/A” provided either only human or only animal stable carbon isotopic data.

Stable carbon isotope data provide additional insight into this process. Notably, *δ*^13^C values consistent with C_4_ plant consumption are systematically first identified in animals and then in humans only in later phases within diachronic contexts dated between 2500 and 1700 BCE (Supplementary Table S1, Fig. 5A). This pattern suggests that millet was initially incorporated as animal fodder and only subsequently integrated into human diets. After 1900/1700 BCE, a primary C_4_ consumption by humans or a synchronous use by humans and animals is attested whenever both human and animal data are available, which is, however, seldom in this dataset. The chronological pattern observed in the consumption of millet primarily by animals or by humans is not associated with a specific spatial distribution (Fig. 5B). This means that the fact that either humans or animals were first to consume C_4_ plants varies over time but is not specific to any geographical location. At sites where only human remains are available and at sites where both humans and animals present elevated *δ*^13^C values, it cannot be excluded that elevated *δ*^13^C values from human remains derive from the consumption of animals consuming millet (or affected by other C_4_ plants or arid/saline conditions) instead of a direct consumption by humans. This has no impact on the dispersion pathway presented in this paper but needs to be considered when investigating how millet was introduced into past agricultural systems.

**Fig. 6:**
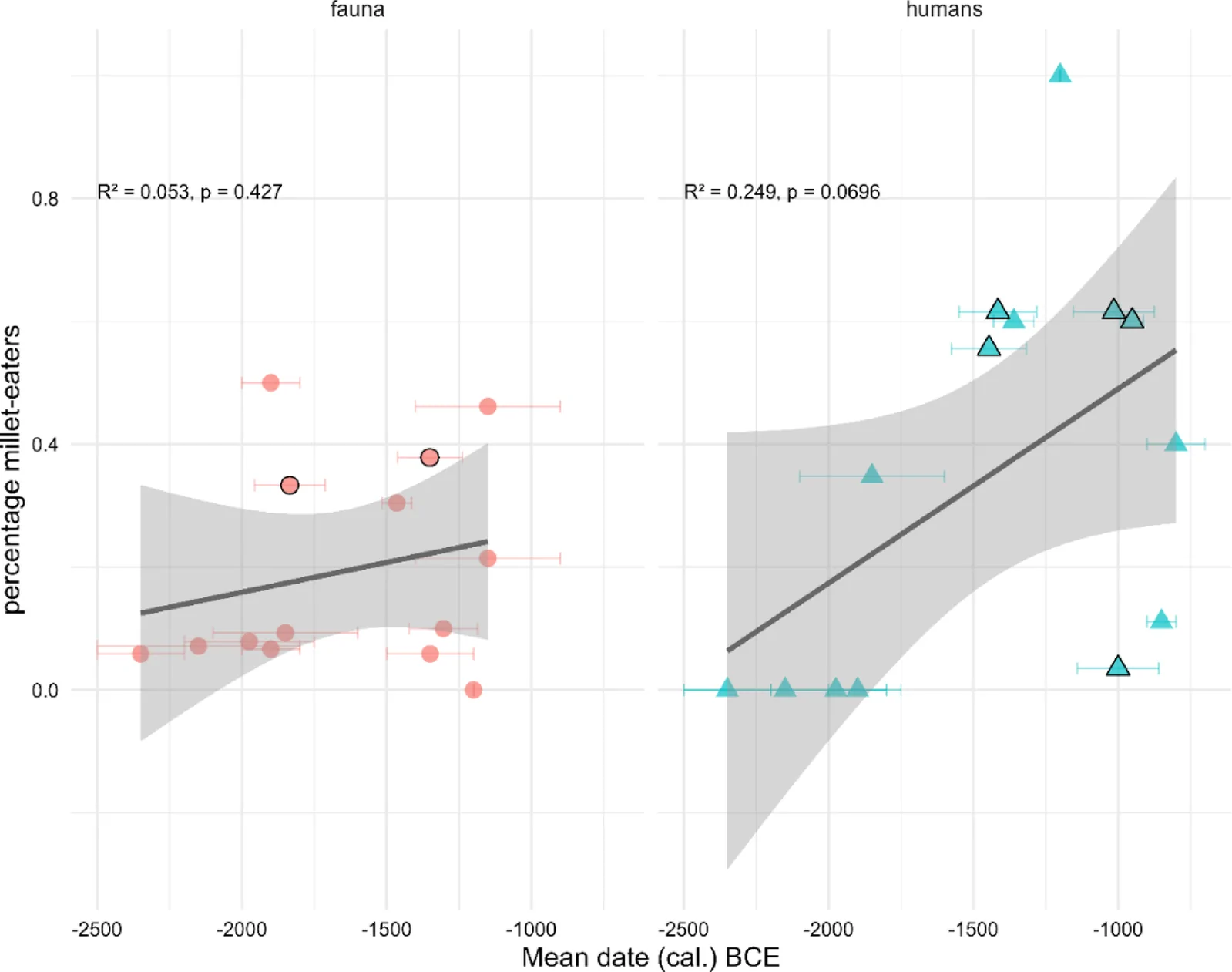
Linear model showing the temporal evolution of the percentage of C_4_-eaters among humans and animals at each site with a sample size of n > 8. Sites providing both human and animal samples appear on both plots accordingly. Dots with a black contour highlight the evidence associated with a radiocarbon-date. The horizontal error bars represent one standard deviation (SD) for the radiocarbon-dated evidence and the total extent of the range for archaeologically determined chronologically. The linear models were conducted in the R software 4.3.2. ^87^ using the *lme4* package ^85^. The selection of sites with sample sizes of n > 8 shall represent the most reliable results ^86^, but results at including sites with smaller sample sizes can also be found in Supplementary Fig. S5. The detailed results are listed in Supplementary Table S1.

The isotopic evidence further indicates that the onset of millet adoption within the study area did not necessarily correspond to population-wide dietary change. At most sites, less than 40% of the analysed humans or animals exhibit isotopic evidence for C_4_ plant consumption (Fig. 6, Supplementary Table S1, Supplementary Fig. S4), suggesting that millet consumption was likely restricted to specific individuals or subgroups rather than reflecting widespread community-level adoption ^83,84^. Higher proportions of C_4_ consumers tend to appear mainly after 1500 BCE, which is consistent with the archaeobotanical evidence and may indicate a more systematic cultivation and consumption during this phase. However, this development is neither linear (R^2^ of 0.05 for animals and of 0.25 for humans at sites with n > 8) nor statistically significant (*p*-values of 0.43 for animals and of 0.07 for humans at sites with n > 8) (Fig. 6); and low levels of C_4_ consumption continue to be observed in later periods (Fig. 6; see also Supplementary Fig. S5 for the linear models including sites with small sample sizes) ^85–87^. The only significant *p*-value (of 0.005) is attested for humans when including all sites (including n < 5, see Supplementary Fig. S5a), yet this is not representative due to the small R^2^ value (of 0.20) and the small sample sizes. Sites with proportions of C_4_ consumers above 50% are distributed across the study area without a clear spatial pattern (Supplementary Fig. S4).

Overall, the available evidence suggests that millet was originally often consumed in a selective or secondary way and was not immediately adopted as a staple crop at the community level. This may explain why the earliest evidence for millet consumption is rather visible via protein biomarkers ^32^ and less so in the isotopic records, which require more substantial C_4_ dietary intakes to be detectable. In addition, the delayed appearance of millet in certain regions, such as Anatolia and parts of southern Greece ^33^, further highlights the complexity and regionally differentiated nature of the adoption process.

The association of millet with specific cultural identities or socio-economic practices has been proposed in previous studies (e.g., ^10,33,42^). Its limited consumption or presence among particular individuals may reflect differences in dietary practices and/or population mobility ^88–90^, while the potential role of environmental and climatic factors in shaping these patterns remains to be further explored.

### Mobility, connectivity, and millet dispersal

Against this background, the rapid spread of millet across large parts of Europe during the later second millennium BCE is consistent with increasing interregional mobility and connectivity linking the western Eurasian steppe with Central and Southeastern Europe ^91–93^. Such networks likely facilitated the transmission of crops alongside other technologies and practices, providing a broader structural context for the dispersal of millet.

Recent genetic evidence provides a broader comparative parallel for large-scale mobility across Eurasia. Genetic studies indicate that the DOM2 lineage of modern domestic horses originated in the western Eurasian steppes and spread rapidly after ca. 2200 BCE ^91,94^, indicating that extensive continental-scale mobility networks were already established along this axis by the late third millennium BCE. The geographical direction of this expansion broadly overlaps with the later spread of millet, potentially following major river corridors such as the Danube.

To conclude, the chronological patterns identified in this study do not support a simple stepwise expansion between regions. Instead, the modeled onset ranges suggest broadly overlapping appearances of directly dated millet grains during the mid–second millennium BCE, pointing to a rapid and spatially diffuse introduction across multiple interconnected regions. Although the Pontic Steppe currently provides the earliest securely dated evidence, the overlapping chronological ranges highlight both the limitations of the available dataset and the likelihood that millet dispersal was embedded within wider networks of mobility and exchange rather than following a single clearly defined route.

In particular, at sites where the number and proportion of C_4_-eaters are very low, sometimes reduced to a single individual, it is likely that such individuals with locally exceptional C_4_ dietary intake represent people of foreign origin, who either kept the habit of millet/C_4_ consumption after their arrival or still exhibit the dietary signal of their place of origin in their tissue due to long remodeling times of some body tissues ^27^. This would suggest that millet spread could have been triggered by the movement of single individuals within specific contexts instead of large-scale migration and demic diffusion. However, other proxies providing more direct evidence of a foreign origin, such as strontium isotopes or genetics, are needed to confirm such hypothesis, since diverging diet is only potential and no direct evidence for mobility ^95^.

It should also be noted that the currently limited archaeobotanical evidence and number of direct radiocarbon dates from regions further east, including parts of the Eurasian Steppe and areas around the Ural Mountains, may partly reflect research gaps rather than a true absence of millet.

Finally, in the present study, directly dated millet remains from contexts previously attributed to the Neolithic in North Macedonia, Ukraine, and Serbia yielded later radiocarbon dates in the present study. This finding further underlines the importance of direct radiocarbon dating, particularly in cases where millet is reported from early archaeological contexts.

## Methods

The archaeobotanical and isotopic review encompasses a broad geographical area extending from the Adriatic Sea to the eastern coast of the Caspian Sea and from northern Ukraine to Crete (Fig. 1). To facilitate the description and presentation of the available evidence across this large study area, broad geographic groupings are used for analytical purposes rather than strict ecological, cultural, or political boundaries.

Isotopic data from neighboring regions, including northwestern Ukraine, the northern Near East, the Caspian lowland desert, and the eastern Caspian littoral, are also considered for broader comparative purposes.

### Radiocarbon dating of archaeobotanical material

To the best of our knowledge, all published direct AMS radiocarbon dates on charred broomcorn millet grains from the regions of interest were compiled (Data S1, see also ^96^). The dataset spans from the earliest occurrences in the mid–second millennium BCE to the latest directly dated millet grains in the early first millennium BCE. In total, the dataset includes 70 direct AMS radiocarbon dates on single charred millet grains, comprising 16 new measurements (Table 1) and 54 previously published determinations.

Only directly AMS-dated single millet grains were included in the dataset. Context-dated remains, seed impressions, and samples with uncertain botanical identification were excluded in order to ensure chronological reliability. Some indirectly dated millet finds were discussed in the article but were not included in the Bayesian model. This is because the radiocarbon date relates to the archaeological context or to another material rather than to the millet grain itself. More specifically, a concentration of millet grains reported from Tabakoni, in present-day Georgia ^34^, was associated with a wood fragment radiocarbon-dated to 1877–1686 cal BCE (95.4%). Since the millet grains themselves were not directly dated, this date was treated as indirect evidence and was not included in the Bayesian model, which includes only AMS dates obtained directly from single millet grains. Therefore, we cannot be certain that the grains actually belong to the same period. The resulting dataset represents a comprehensive compilation of all currently available direct AMS radiocarbon determinations on charred millet grains from the study regions and period.

The new AMS radiocarbon measurements were obtained from charred single grains recovered at ten archaeological sites in Greece, Türkiye, Bulgaria, North Macedonia, Serbia, and Ukraine. AMS analyses were carried out at the Vilnius Radiocarbon Laboratory of the Center for Physical Sciences and Technology (FTMC, Vilnius, Lithuania) and at the Leibniz Laboratory for AMS Dating and Isotope Research (Kiel, Germany). All samples were pretreated using the standard acid–base–acid (ABA) protocol, converted to graphite, and measured using accelerator mass spectrometry according to the analytical procedures of each laboratory. At FTMC, graphitisation was performed using an AGE-3 system (Ionplus AG), and AMS measurements were conducted on SSAMS and LEA accelerator systems. Samples processed at the Leibniz Laboratory were combusted in sealed quartz tubes, reduced, and graphitised for measurement in a HVEE 3 MV Tandetron 4130 accelerator. Laboratory codes and full analytical results are reported in the consolidated radiocarbon dataset.

All radiocarbon determinations, both newly obtained and previously published, were recalibrated uniformly using OxCal v4.4.4 ^97,98^ and the IntCal20 calibration curve ^99^.

### Bayesian chronological modeling

To account for chronological uncertainty in the radiocarbon dataset, Bayesian chronological modeling was implemented in OxCal v4.4.4 ^97,98^ using the IntCal20 calibration curve ^99^.

For each geographic region, directly dated millet grains, both published and newly obtained, were treated as unordered dated events within a single uniform Phase delimited by start and end Boundaries. The end Boundary was included to define the closing limit of the uniform Phase and was not interpreted as the end of millet cultivation or consumption. Only the posterior distribution of the start Boundary was used to estimate the earliest probable appearance of directly dated millet within each region.

The regional framework used for modeling broadly follows previous studies on the dispersal of millet across Eurasia, particularly the macro-regional structure proposed by Filipović et al. ^20^ for Europe and Martin et al. ^15^ for the Caucasus. These groupings are employed as heuristic analytical units for comparative chronological modeling and do not imply strictly homogeneous ecological, cultural, or political zones. Within this framework, sites assigned to the Pontic Steppe grouping broadly correspond to the northwestern Black Sea zone extending from present-day Ukraine towards the lower Danube, following the regional structure proposed by Filipović et al. ^20^ and related studies ^4^. The dataset was further expanded through the inclusion of the recently published site of Dobrovăț ^100^, located in northeastern Romania, and assigned here to the Pontic Steppe grouping for analytical purposes (Supplementary Methods S1). The Caucasus is treated here as a single macro-region encompassing both the northern and southern slopes of the Caucasus mountain system, following approaches adopted in previous archaeobotanical and regional studies ^15,101^. Groupings corresponding to the Carpathian Basin and Southeastern Europe also broadly follow the regional framework proposed by Filipović et al*.* ^20^*,* although they are restricted here to sites included in the present dataset. Newly obtained data from sites in Greece, Bulgaria, and Serbia were incorporated into the corresponding groupings. In addition, sites from present-day Türkiye were grouped within an additional analytical unit referred to here as Anatolia.

Each region was modeled independently using an identical model structure to produce comparable estimates of first appearance across the study area. No *priori* chronological order was imposed between regions, allowing regional onset estimates to be compared without predefining a dispersal pathway. Chronological interpretation in this study relies on posterior ranges derived from Bayesian modeling rather than on individual calibrated radiocarbon dates alone.

The precision of the resulting start estimates varies between regions depending on the number of dated grains, the number of archaeological sites represented, and the chronological distribution of available determinations. In some cases, multiple radiocarbon dates derive from the same site or from closely related stratigraphic contexts. In the modeling framework, all available determinations were included within the same Phase in order to estimate the most probable onset of millet presence at a regional scale. No date combinations were applied, as the aim of the model was to estimate regional onset based on the full set of available direct determinations rather than to construct site-specific chronologies.

Several newly obtained AMS radiocarbon determinations yielded dates substantially later than their associated archaeological contexts and fell outside the predefined chronological scope of the model. These results indicate that the grains represent later intrusions into earlier archaeological deposits. They were therefore excluded from the regional onset models but are reported in Table 1.

Details of the Bayesian chronological modeling and OxCal scripts are provided in Supplementary Methods S1.

Archaeobotanical modeling and isotopic evidence were evaluated independently, as the two datasets represent different processes (crop presence versus dietary consumption) and differ in chronological resolution.

### Review of isotope data

*Principles* – Millet uses a C_4_ photosynthetic pathway and discriminates less against the heavier carbon isotope ^13^C than C_3_ plants during carbon capture from the atmosphere ^102^. This means that millet and other C_4_ plants present higher *δ*^13^C values (expressing the ratio of ^13^C/^12^C isotopes in ‰) compared to C_3_ plants ^103^. And because the isotope composition of the ingested food is transferred to the body tissues of the consumer, millet consumers have substantially higher *δ*^13^C values in their tissues compared to people on a C_3_ diet ^27,104^. Stable carbon isotope analysis of bioarchaeological human or animal remains – mostly combined with nitrogen isotope analysis – is thus a powerful tool to trace C_4_ plant consumers down to the individual in the past. Published *δ*^13^C data overall provide a good complementary information to archaeobotanical remains, since it directly informs on the presence and proportions of millet in human and animal diet. In the easternmost, southernmost, and coastal regions of this research area, however, wild C_4_ plants, arid conditions and the influence of marine sources are important factors that may have resulted in equally elevated *δ*^13^C values in human and animal remains, without being evidence for millet consumption ^103,105^. The identification of millet grains at a given site and the comparison with *δ*^15^N values therefore help interpret elevated *δ*^13^C values as actual evidence for millet intake versus the ingestion of other C_4_ plants or marine food, or the influence of arid conditions ^27,105^. This method nevertheless has clear limits due to the equifinality of isotope data and the difficulty to disentangle the various possible reasons for elevated *δ*^13^C values. This is the reason why we differentiate clear carbon isotopic identifications of millet diet (based on contextual data) presented in the main text, from unclear elevated *δ*^13^C values (that may be either millet or another factor) presented in the supplementary materials.

*Identifying millet-consumers*—In enamel or bone apatite, *δ*^13^C values above − 9‰ generally indicate a mixed diet of C_3_ and C_4_ plants, whereas − 4‰ and above represent the end-member of a pure C_4_ diet ^106,107^. As for bone and dentine collagen, a carbon isotopic threshold at − 18‰ has been commonly defined to identify the introduction of substantial C_4_ plants within a C_3_ diet ^108^, while *δ*^13^C values above − 12‰ represent a pure C_4_ diet ^106,107^. However, plant *δ*^13^C values vary across the globe depending on their geographical location and the environmental/climatic settings in which they grow ^105,109,110^. We therefore used the ecologically dynamic threshold values defined by Depaermentier et al. ^47^ for C_4_-plant consumer identification across Europe. The eastern edge of the research area has no such environmentally adjusted threshold yet, the estimations are based on published data and consider local ecological settings. This allowed for an isotopic determination of the presence/absence (yes/no in Data S2) of millet in the diet of each human or animal included in this study. In some cases, a doubt remained regarding the interpretation of elevated *δ*^13^C values due to the early date of the sample and/or to the plausible role of other factors leading to similarly enriched *δ*^13^C values (e.g., ^105^). Such cases are marked as “unclear” or as “elevated but not millet” in the dataset (Data S2).

*Data collection* – Isotope data were collected until January 2026 from the literature ^15,28,30,31,35–41,43–46,48–50,52–57,59,65,66,68,69,74,76–78,111–172^ using open access online databases such as IsoArch ^173,174^, the North Central Asia isotopic database and the North-Eastern Europe and Northern Asia isotopic dataset of bioarchaeological samples (NEENA) ^175^ datasets from previous large-scale reviews ^7,42^, and original literature. This represents a total of 3512 individuals (and five population means), including 1358 animals and 2213 humans (Data S2, see also ^176^). In total, 3350 entries represent collagen data and 815 are apatite data. Multiple apatite entries often belong to the same animal, due to micro-sampling, but they were not averaged, as they represent different moments of dietary intake and are not replicates of a single sample. 62 human individuals provided data from both collagen and apatite. In this case, it is worth stressing that apatite is more sensitive to the isotopic composition of the consumed plants such as millet, whereas collagen is affected by the protein part of the diet and might thus more strongly reflect the influence of animal-based food ^177^. They hence provide a complementary insight into dietary habits, which can help disentangle ambiguity in the omnivorous human diet.

*Spatial framework* – The dataset was divided into the main regions of investigation as described above, i.e., the Carpathian Basin (n = 64 entries), Anatolia (n = 256 entries), Southeastern Europe / Balkans (n = 1457 entries), Pontic steppe (n = 1035 entries), and the Caucasus (n = 1067 entries). Data from neighbouring regions were included for comparison: Eastern Caspian coast (n = 22 entries), Caspian lowland desert (n = 16 entries), Western Russia (n = 77 outside the actual research area), the northern Near East (n = 90 entries), and Northwestern Ukraine (n = 39 entries). A total of 271 archaeological sites are included within these ten regions.

*Chronological framework* – To consider the chronological depth of the earliest potential millet introduction within the various regions, the core of the samples was collected for the period between 2200/2000 and 1200 BCE. This period was targeted due to the expected chronology derived from previous archaeobotanical and isotopic research, coupled to radiocarbon dates (e.g. ^7,11,20,30,31,42,84^). Data from the earliest phase of this period, e.g., 2200–1700 BCE, was particularly collected in order to verify the absence of millet in human or animal diets before the expected dates. The research area moreover includes regions in which factors other than millet can increase the *δ*^13^C values of body tissues, e.g., the availability of wild C_4_ plants, the access to marine food, and/or arid conditions ^44,105,178–180^. Carbon isotope data from periods that surely predate the arrival of millet (c. 6000 to 2000 BCE) were therefore added to the dataset to assess the potential isotopic impact of these other factors in each region.

*Chronological uncertainty* – Most samples from which stable isotope data derived were not radiocarbon dated and their assigned chronology derived from the archaeological context. This often provides particularly wide ranges, with nearly a third (i.e., n = 1183) of the data associated with a chronological span of at least 500 years, including 666 with a chronological span between 800 and 2000 years (Supplementary Fig. S6). Published data showing a chronological span of over 2000 years were not included in the study. This lack of chronological precision reduces the possibility of comparing these data with radiocarbon-dated archaeobotanical remains. Nevertheless, almost half of the sample (n = 1951) shows a chronological span of between 110 and 350 years, and 274 data show even less. This does not account for the data for which a single date instead of a range was available, providing a misleading impression of chronological precision. A minimum, maximum and mean date was furthermore allocated to each sample based on the AMS date (cal BCE) when available, or on the archaeologically defined chronological span (BCE). In this case, the related analyses were run both with and without the samples with a chronological uncertainty (or range) larger than 350 years. To define the earliest evidence for millet consumption (or other elevated *δ*^13^C values) at each site, the AMS date was preferred to the archaeological date when available. Regarding archaeological dates, the sample with the narrowest range among the oldest was selected for the estimation of the earliest evidence for millet intake.

## Supplementary Information

Below is the link to the electronic supplementary material.

Supplementary Material 1

Supplementary Material 2

Supplementary Material 3

Supplementary Material 4

## Data Availability

The datasets generated and/or analysed during the current study are provided in the Supplementary Information file and associated supplementary datasets (Data S1 and Data S2, Supplementary Table S1). The archaeobotanical and radiocarbon dataset used in this study (Data S1) is also publicly available on the open-access repository *Zenodo* via https://doi.org/10.5281/zenodo.20426946 (see reference ^96^). The full compilation of published stable carbon isotope data used in this isotopic review (Data S2) is also publicly available on the open-access repository *Zenodo* via https://doi.org/10.5281/zenodo.20425401 (see reference ^176^).
