# Supplementary material for: The initial pathways of broomcorn millet (Panicum miliaceum) into Europe based on AMS dates and stable carbon isotope data
Source: Sci Rep. 2026 Sep 4;16:34554. doi: 10.1038/s41598-026-70250-w (PMC13713580; doi:10.1038/s41598-026-70250-w)
Supplement: Supplementary file 1 [file 41598_2026_70250_MOESM4_ESM.pdf]

Supplementary Information:

The initial pathways of broomcorn millet (Panicum miliaceum) into Europe based on AMS dates and stable carbon isotope data

Kyriaki Karanikola, Margaux L. C. Depaermentier, Tom Maltas, John Meadows, Dragana Filipović, Soultana-Maria Valamoti, Michael Kempf, Andrew Fairbairn, Michaela Vychronová, Goce Naumov, Sophia Koulidou, Clémence Pagnoux, Hanna Aleksandrova, Tzvetana Popova and Giedrė Motuzaite-Matuzeviciute.

Corresponding authors:

Kyriaki Karanikola — kyriaki.karanikola@if.stud.vu.lt

Margaux L. C. Depaermentier — margaux.depaermentier@if.vu.lt

Giedrė Motuzaite-Matuzeviciute — giedre.motuzaite@gmail.com

Contents:

- Supplementary Figures *(Supplementary Figs. S1-S6)*
- Supplementary Methods S1. Bayesian chronological modelling
- OxCal script

Associated datasets (uploaded separately):

Data S1. Archaeobotanical dataset of directly dated broomcorn millet grains (.xlsx)

Data S2. Dataset of published stable carbon isotope values from collagen and apatite samples of human and animal remains, including references (.xlsx)

Supplementary Table S1. Overview of the number and proportions of human and animal individuals with evidence for C_4 plant consumption at each site (.xlsx)

Supplementary Figures:

Supplementary Fig. S1. Carbon and nitrogen isotope values from human and animal collagen samples presented by region. The grey shaded area represents the range of $\delta^{13}C$ values between -18 and -16‰, which are considered potential thresholds for C_4 diet identification across the research area [57]. Detailed results with associated chronology can be found in Data S2.

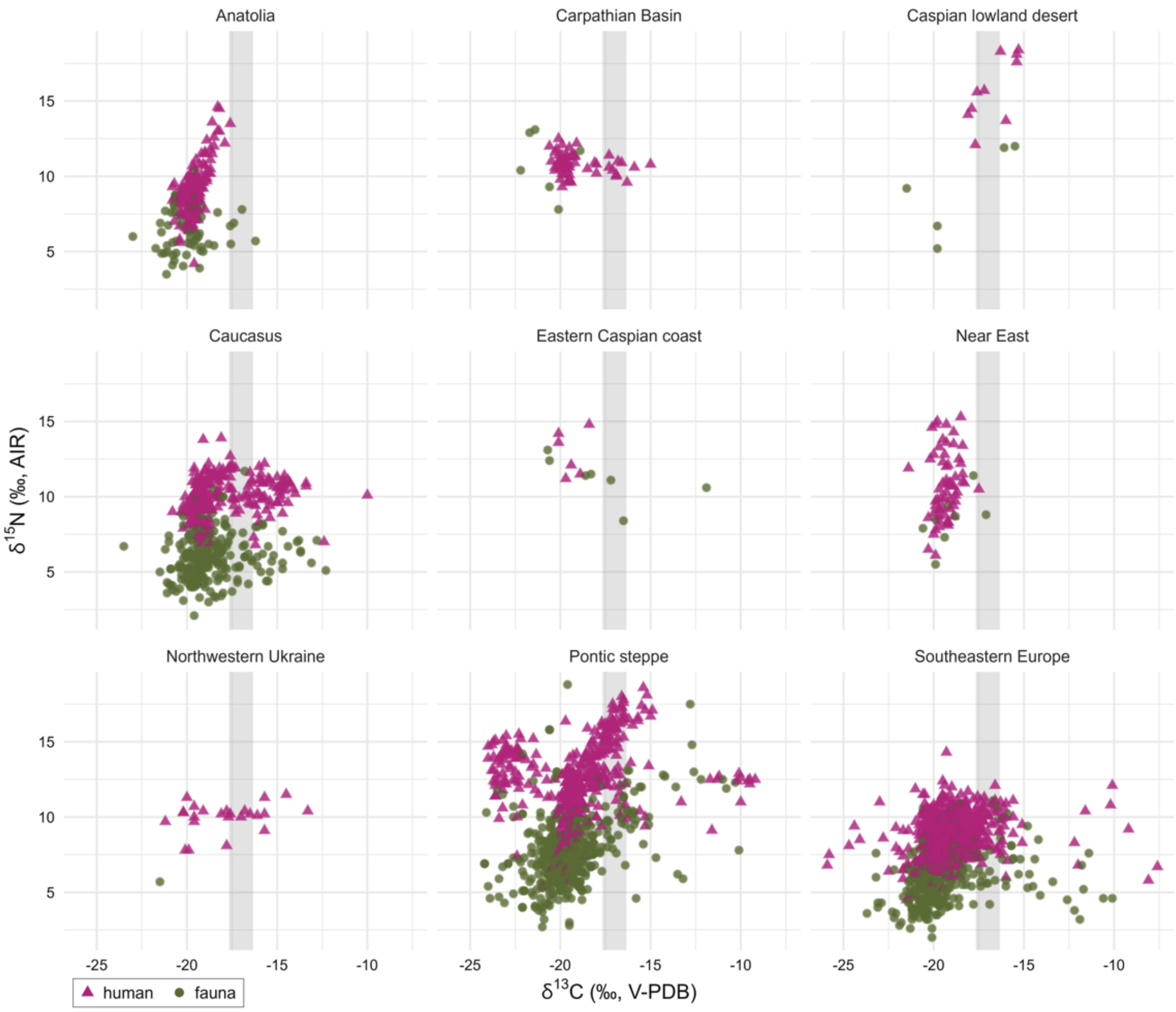

Supplementary Fig. S2. Carbon isotope values from human and animal bone and enamel apatite samples presented by region. Detailed results with associated chronology can be found in Data S2.

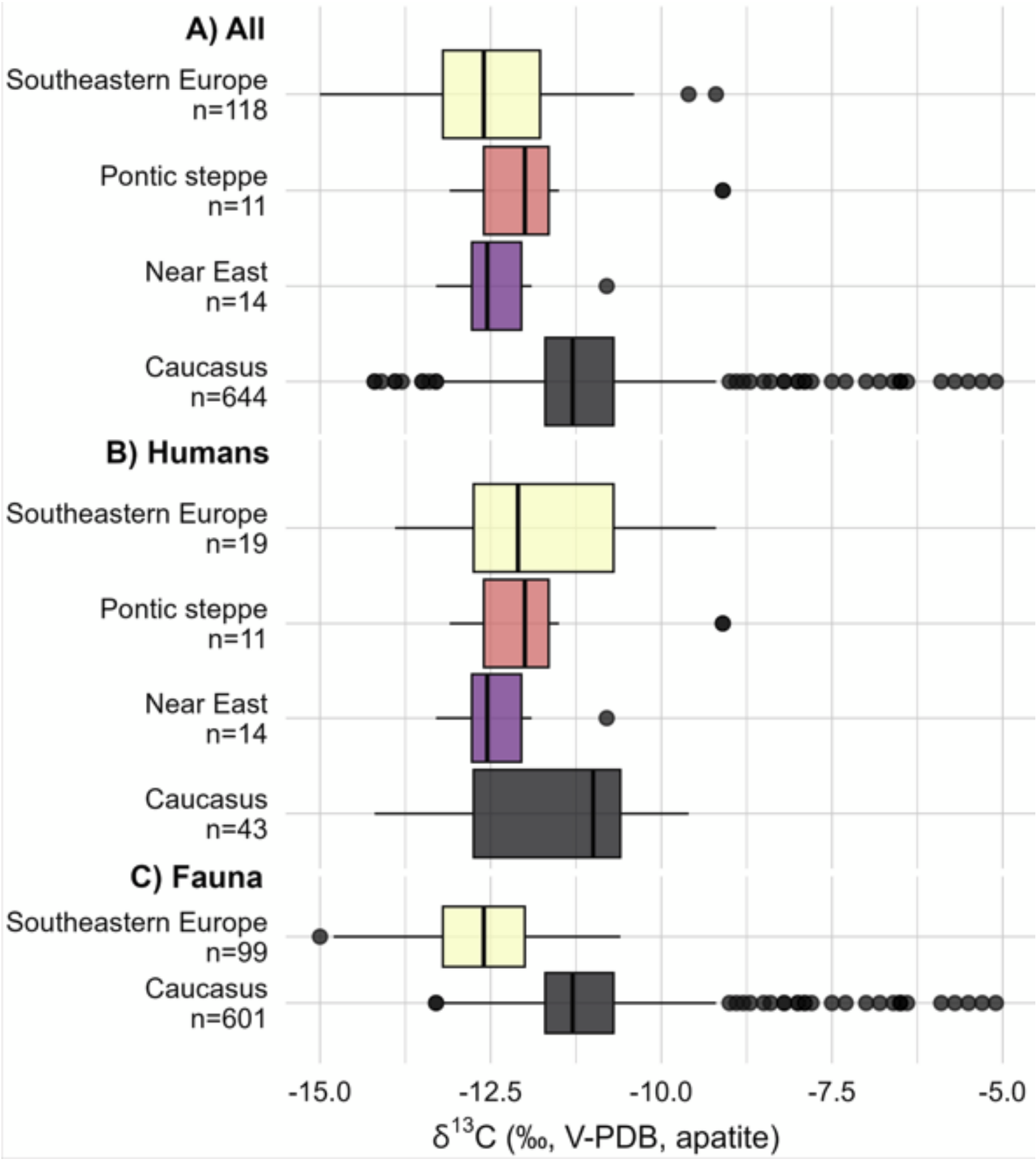

Supplementary Fig. S3. **Distribution of archaeological sites with elevated collagen or apatite δ^{13}C values.** Colored signatures reflect the mean date (cal) BCE associated with the elevated δ^{13}C values. Grey triangles represent evidence for C_3 diet only. The lower panel illustrates the distribution of these earliest dates across longitude, with the color showing the mean date and error bars showing the dating range.

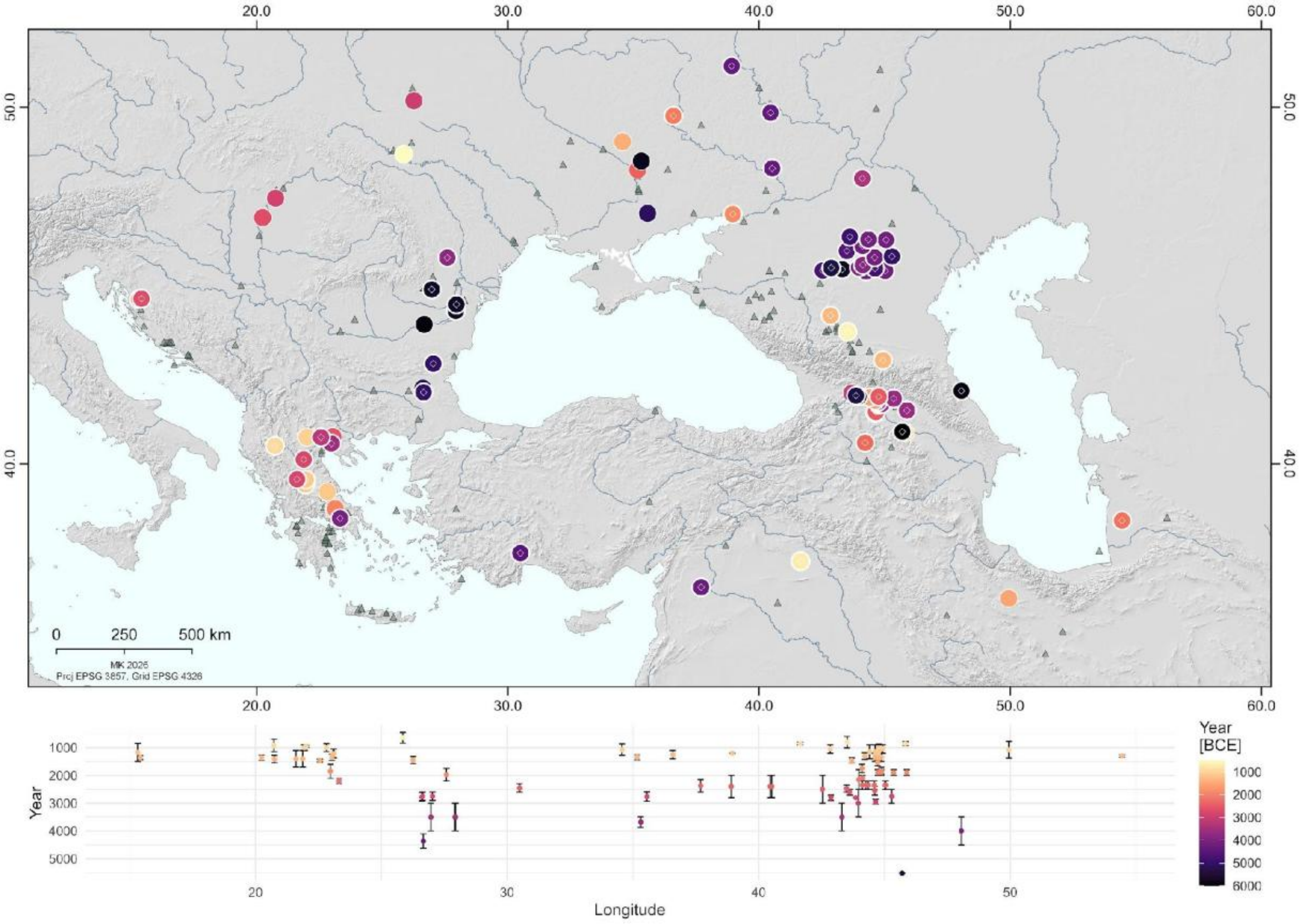

Supplementary Fig. S4. Spatial distribution of the sites with n>5 at which various proportions of C_4 consumers are isotopically identified among humans or animals. The chronological dimension of these sites is depicted in Supplementary Fig. S5.

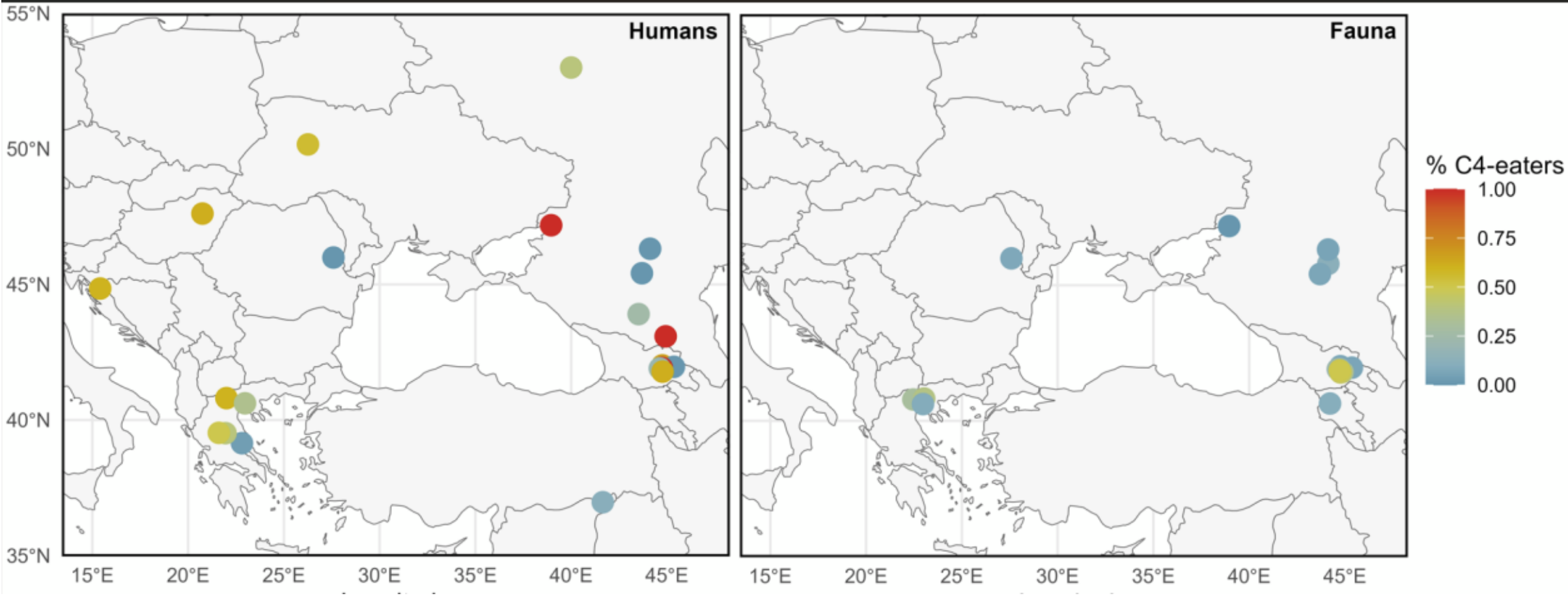

Supplementary Fig. S5. Linear model showing the temporal evolution of the percentage of C_4-eaters among humans and animals at the various sites. A) Results including all sites, regardless of the sample size (including n<5). **B)** Results including only sites with n>5 onwards. The selection of sites with sample sizes of n>8 shall represent the most reliable results [86] and are depicted in Fig. 6. The horizontal error bars represent one standard deviation (SD) for the radiocarbon-dated evidence and the total extent of the range for archaeologically determined chronologies. The linear models were conducted in the R software 4.3.2 [87] using the *lme4* package [85]. The detailed results are listed in Table S1.

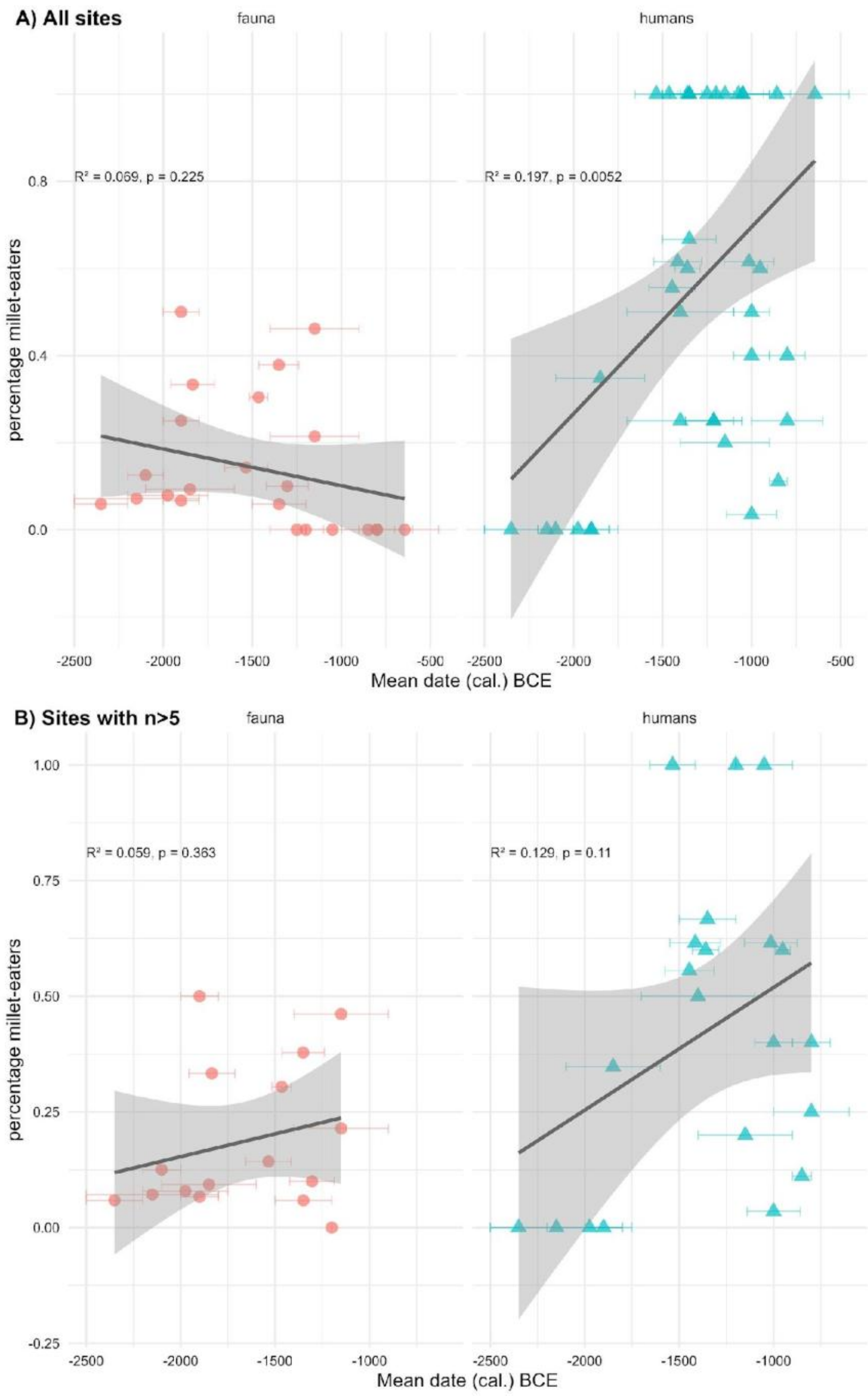

Supplementary Fig. S6. Mean date (BCE/CE) compared to the chronological span of each sample considered in the isotopic review. The chronological span represents the span between the minimum and maximum date provided for the archaeological or radiocarbon date for each sample.

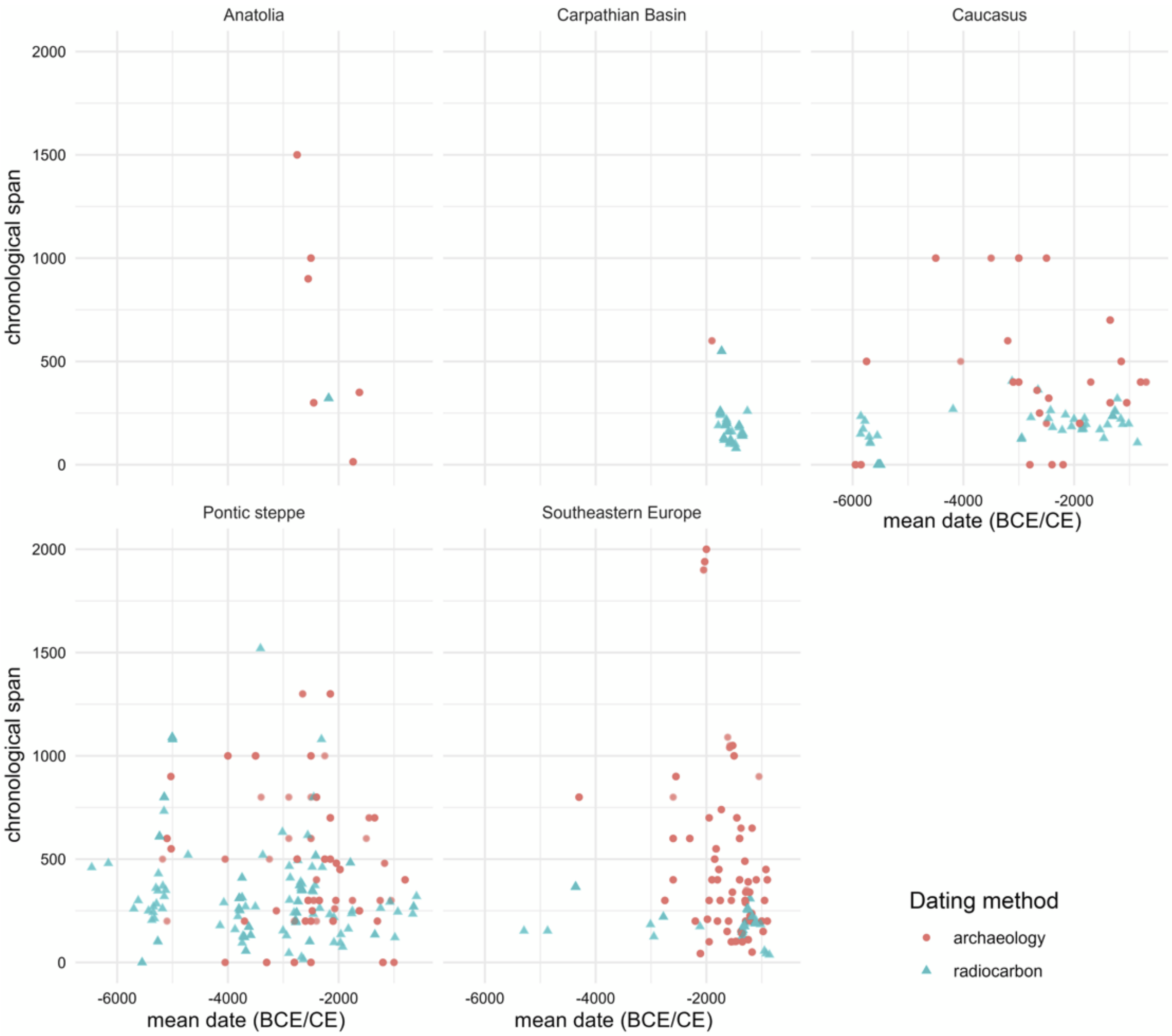

Supplementary Methods S1. Bayesian chronological modelling

To account for chronological uncertainty in the radiocarbon dataset, Bayesian chronological modeling was undertaken in OxCal v4.4.4 [97] using the IntCal20 calibration curve [99]. For each macro-region, directly dated broomcorn millet grains were treated as unordered dated events within a single uniform Phase delimited by start and end Boundaries. No fixed calendar dates or informative priors were assigned to either Boundary; both were estimated from the included radiocarbon determinations. The end Boundary was required to define the closing limit of the uniform Phase and was not archaeologically interpreted. Only the posterior distribution of the start Boundary was used to estimate the earliest probable appearance of directly dated millet. The standard uniform Phase model was selected because no independent archaeological evidence was available to justify imposing an exponential or other specific temporal distribution on the dated events.

All regions were modeled independently using an identical model structure in order to produce directly comparable estimates of first appearance across the study area. No *a priori* chronological order was imposed between regions, allowing regional onset estimates to be compared without predefining a dispersal route. Chronological interpretation therefore relies on posterior modeled ranges rather than on individual calibrated radiocarbon dates alone.

The precision of the resulting start estimates varies between regions according to the number of dated grains, the number of archaeological sites represented, and the chronological distribution of available determinations. Differences between regional start boundaries were subsequently used to evaluate the relative timing of millet appearance across the study area.

Not all newly obtained radiocarbon dates generated in the present study were included in the Bayesian models. Dates identified as intrusive or substantially later than the archaeological contexts from which they derived were excluded from modeling but are reported in Table 1 and Data S1.

Pontic Steppe

Within this framework, the Pontic Steppe grouping includes the sites assigned to the *north_pontic* category, as defined by Filipović et al. (2020) [20], which broadly corresponds to the northwestern Black Sea zone. This area includes both steppe and forest-steppe environments extending from present-day Ukraine towards the lower Danube [19]. The present dataset is further expanded by the inclusion of the recently published site of Dobrovăț [100], which was assigned to this grouping due to its location within the forest-steppe zone of the Pontic Steppe.

Included radiocarbon dates:

Model_region	Site name	Lab code	14C Age (BP)	Error (±BP)
Pontic_Steppe	Dobrovat	Beta-678284	2690	30
Pontic_Steppe	Dobrovat	Beta-678285	2560	30
Pontic_Steppe	Dobrovat	Beta-678288	2740	30
Pontic_Steppe	Dobrovat	Beta-678290	3110	30
Pontic_Steppe	Dobrovat	Beta-678293	2850	30
Pontic_Steppe	Dobrovat	Beta-678295	2760	40
Pontic_Steppe	Dobrovat	Beta-678296	2780	30
Pontic_Steppe	Dobrovat	Beta-678299	2800	30
Pontic_Steppe	Dikiy sad	Poz-103214	2905	35
Pontic_Steppe	Dikiy sad	Poz-105274	2829	35
Pontic_Steppe	Vinogradnyi sad	KIA-53658	3090	35
Pontic_Steppe	Vinogradnyi sad	Poz-103215	3160	30
Pontic_Steppe	Vinogradnyi sad	Poz-105275	3275	35

Modelled start boundary estimates:

- **68.3% probability:** 1614–1494 BCE / 1489–1479 BCE
- **95.4% probability:** 1724–1446 BCE

Agreement indices:

- **Amodel:** 104.3
- **Aoverall:** 104.5

Caucasus

In the present study, the Caucasus is treated as a single macro-region encompassing both the northern and southern slopes of the Caucasus mountain system, following approaches adopted in archaeobotanical and regional studies of the area (e.g., [15, 101]).

Included radiocarbon dates:

Model_region	Site name	Lab code	14C Age (BP)	Error (±BP)
Caucasus	Namcheduri	Poz-66777	3150	35
Caucasus	Gudabertka	Poz-66771	2815	30
Caucasus	Grakliani gora	Poz-51404	2935	30
Caucasus	Gegharot	Poz-63429	2995	30
Caucasus	Tsaghkahovit	Poz-66779	2945	30
Caucasus	Chishkho	Poz-82836	3000	30
Caucasus	Chidgom	Poz-82835	2870	30
Caucasus	Guamsky grot	GrA-65338	2835	35
Caucasus	Kabardinka2	Poz-82838	2950	30

Modelled start boundary estimates:

- **68.3% probability:** 1466–1411 BCE / 1406–1316 BCE
- **95.4% probability:** 1593–1276 BCE

Agreement indices:

- **Amodel:** 86.0
- **Aoverall:** 85.5

Carpathian Basin

The Carpathian Basin grouping broadly follows Filipović et al. (2020) [20], although it is restricted here to sites included in the present dataset.

Included radiocarbon dates:

**Model_region**	**Site name**	**Lab code**	**14C Age (BP)**	**Error (±BP)**
Carpathian_Basin	Mackovac crisnjevi	Poz-104900	3120	70
Carpathian_Basin	Mackovac ostrovi	Poz-104921	3055	35
Carpathian_Basin	Cornesti	MAMS-35587	3048	18
Carpathian_Basin	Cornesti	MAMS-35958	3085	21
Carpathian_Basin	Cornesti	MAMS-35961	3087	22
Carpathian_Basin	Cornesti	MAMS-35963	3068	23
Carpathian_Basin	Cornesti	MAMS-35964	3049	22
Carpathian_Basin	Cornesti	MAMS-35966	3051	32
Carpathian_Basin	Magura buduiasca	OxA-26706	3093	35
Carpathian_Basin	Teleac	Poz-102424	2755	30
Carpathian_Basin	Gomolava	Poz-98013	2630	30
Carpathian_Basin	Gomolava	Poz-98015	2670	30
Carpathian_Basin	Gomolava	Poz-98016	2775	30
Carpathian_Basin	Kalakaca	Poz-100052	2725	30
Carpathian_Basin	Skorenovac	KIA-56391	1965	90
Carpathian_Basin	Skorenovac	KIA-56390	1900	45
Carpathian_Basin	Bosut	Poz-98017	2680	30
Carpathian_Basin	Fajsz 18	OxA-26703	3075	36
Carpathian_Basin	Fajsz 18	OxA-26704	3214	36
Carpathian_Basin	Pecel	KIA-53627	3140	28
Carpathian_Basin	Pecel	Poz-100051	2965	30
Carpathian_Basin	Pecel	KIA-53628	3070	35
Carpathian_Basin	Szazhalombatta	Poz-98088	2770	30

Modelled start boundary estimates:

- **68.3% probability:** 1560–1450 BCE
- **95.4% probability:** 1688–1424 BCE

Agreement indices:

- **Amodel:** 95.5
- **Aoverall:** 95.2

Anatolia

Anatolia was treated as a separate analytical unit corresponding broadly to the geographical area of present-day Türkiye.

Included radiocarbon dates:

**Model_region**	**Site name**	**Lab code**	**14C Age (BP)**	**Error (±BP)**
Anatolia	Sos hoyuk	Poz-66780	2945	35
Anatolia	Sos hoyuk	Poz-66781	2960	35
Anatolia	Troy	FTMC-US70-1	2885	33
Anatolia	Troy	FTMC-US70-2	2959	31

Modelled start boundary estimates:

- **68.3% probability:** 1252–1137 BCE
- **95.4% probability:** 1402–1068 BCE

Agreement indices:

- **Amodel:** 99.7
- **Aoverall:** 103.3

Southeastern Europe

The Southeastern Europe grouping follows the regional framework employed by Filipović et al. (2020) [20], encompassing sites from the Balkan Peninsula south of the Danube, including Greece and southern Serbia. The present dataset expands this regional group by including newly directly dated millet grains from additional sites in Greece, southern Serbia, and Bulgaria.

Included radiocarbon dates:

Model_region	Site name	Lab code	14C Age (BP)	Error (±BP)
Southeastern_Europe	Nova nadejda	FTMC-SW48-2	2804	32
Southeastern_Europe	Archontiko	Poz-144413	2920	35
Southeastern_Europe	Archontiko	Poz-144414	3135	35
Southeastern_Europe	Assiros	HD-25509	3071	12
Southeastern_Europe	Rema xydias	FTMC-OG79-1	3046	31
Southeastern_Europe	Rema xydias	FTMC-OG79-2	3047	34
Southeastern_Europe	Skala sotiros	FTMC-SW48-6	3049	34
Southeastern_Europe	Skala sotiros	Poz-144410	3130	35
Southeastern_Europe	Skala sotiros	Poz-144411	3150	35
Southeastern_Europe	Skala sotiros	Poz-144412	3115	35
Southeastern_Europe	Hisar	Poz-105052	2965	35
Southeastern_Europe	Hisar	Poz-98085	2920	35
Southeastern_Europe	Piljakovac krzince	KIA-56389	3060	30
Southeastern_Europe	Ranutovac	KIA-56387	2560	29
Southeastern_Europe	Ranutovac	KIA-56388	2630	45
Southeastern_Europe	Svinjaricka cuka	KIA-56392	3166	28
Southeastern_Europe	Svinjaricka cuka	KIA-56393	3190	40

Modelled start boundary estimates:

- **68.3% probability:** 1526–1446 BCE
- **95.4% probability:** 1608–1426 BCE

Agreement indices:

- **Amodel:** 110.8
- **Aoverall:** 110.5

Supplementary Model Code.

The complete OxCal v4.4.4 Chronological Query Language code used in this study is provided below to ensure transparency and reproducibility. Users may copy the code into the OxCal input window, save it as an OxCal project file, and run the model. OxCal is freely available at https://c14.arch.ox.ac.uk/oxcal/OxCal.html.

```
Options()

{
  Curve="IntCal20";
};

Plot()
{
  Sequence("Pontic_steppe")
  {
    Boundary("Pontic_steppe_start");

    Phase("Pontic_steppe_sites")
    {
      Phase("Dobrovat")
      {
        R_Date("Beta-678284 (Dobrovat)",2690,30);
        R_Date("Beta-678285 (Dobrovat)",2560,30);
        R_Date("Beta-678288 (Dobrovat)",2740,30);
        R_Date("Beta-678290 (Dobrovat)",3110,30);
        R_Date("Beta-678293 (Dobrovat)",2850,30);
        R_Date("Beta-678295 (Dobrovat)",2760,40);
        R_Date("Beta-678296 (Dobrovat)",2780,30);
        R_Date("Beta-678299 (Dobrovat)",2800,30);
      };

      Phase("Dikiy_Sad")
      {
        R_Date("Poz-103214 (Dikiy Sad)",2905,35);
        R_Date("Poz-105274 (Dikiy Sad)",2829,35);
      };

      Phase("Vinogradnyi_Sad")
      {
        R_Date("KIA-53658 (Vinogradnyi Sad)",3090,35);
        R_Date("Poz-103215 (Vinogradnyi Sad)",3160,30);
        R_Date("Poz-105275 (Vinogradnyi Sad)",3275,35);
      };
    };

    Boundary("Pontic_steppe_end");
  };

  Sequence("Carpathian_Basin")
  {
    Boundary("Carpathian_Basin_start");

    Phase("Carpathian_Basin_sites")
    {
```

```
Phase("Mackovac_Crisnjevi")
{
  R_Date("Poz-104900 (Mackovac Crisnjevi)",3120,70);
};

Phase("Mackovac_Ostrovi")
{
  R_Date("Poz-104921 (Mackovac Ostrovi)",3055,35);
};

Phase("Cornesti")
{
  R_Date("MAMS-35587 (Cornesti)",3048,18);
  R_Date("MAMS-35958 (Cornesti)",3085,21);
  R_Date("MAMS-35961 (Cornesti)",3087,22);
  R_Date("MAMS-35963 (Cornesti)",3068,23);
  R_Date("MAMS-35964 (Cornesti)",3049,22);
  R_Date("MAMS-35966 (Cornesti)",3051,32);
};

Phase("Magura_Buduiasca")
{
  R_Date("OxA-26706 (Magura Buduiasca)",3093,35);
};

Phase("Teleac")
{
  R_Date("Poz-102424 (Teleac)",2755,30);
};

Phase("Gomolava")
{
  R_Date("Poz-98013 (Gomolava)",2630,30);
  R_Date("Poz-98015 (Gomolava)",2670,30);
  R_Date("Poz-98016 (Gomolava)",2775,30);
};

Phase("Kalakaca")
{
  R_Date("Poz-100052 (Kalakaca)",2725,30);
};

Phase("Bosut")
{
  R_Date("Poz-98017 (Bosut)",2680,30);
};

Phase("Fajsz_18")
{
  R_Date("OxA-26703 (Fajsz 18)",3075,36);
  R_Date("OxA-26704 (Fajsz 18)",3214,36);
};

Phase("Pecel")
{
  R_Date("KIA-53627 (Pecel)",3140,28);
  R_Date("Poz-100051 (Pecel)",2965,30);
  R_Date("KIA-53628 (Pecel)",3070,35);
};
```

```
    Phase("Szazhalombatta")
    {
      R_Date("Poz-98088 (Szazhalombatta)",2770,30);
    };
  };

  Boundary("Carpathian_Basin_end");
};

Sequence("Southeastern_Europe")
{
  Boundary("Southeastern_Europe_start");

  Phase("Southeastern_Europe_sites")
  {
    Phase("Nova_Nadejda")
    {
      R_Date("FTMC-SW48-2 (Nova Nadejda)",2804,32);
    };

    Phase("Archontiko")
    {
      R_Date("Poz-144413 (Archontiko)",2920,35);
      R_Date("Poz-144414 (Archontiko)",3135,35);
    };

    Phase("Assiros")
    {
      R_Date("HD-25509 (Assiros)",3071,12);
    };

    Phase("Rema_Xydias")
    {
      R_Date("FTMC-OG79-1 (Rema Xydias)",3046,31);
      R_Date("FTMC-OG79-2 (Rema Xydias)",3047,34);
    };

    Phase("Skala_Sotiros")
    {
      R_Date("FTMC-SW48-6 (Skala Sotiros)",3049,34);
      R_Date("Poz-144410 (Skala Sotiros)",3130,35);
      R_Date("Poz-144411 (Skala Sotiros)",3150,35);
      R_Date("Poz-144412 (Skala Sotiros)",3115,35);
    };

    Phase("Hisar")
    {
      R_Date("Poz-105052 (Hisar)",2965,35);
      R_Date("Poz-98085 (Hisar)",2920,35);
    };

    Phase("Piljakovac_Krzince")
    {
      R_Date("KIA-56389 (Piljakovac Krzince)",3060,30);
    };

    Phase("Ranutovac")
    {
```

```
      R_Date("KIA-56387 (Ranutovac)",2560,29);
      R_Date("KIA-56388 (Ranutovac)",2630,45);
    };

    Phase("Svinjaricka_Cuka")
    {
      R_Date("KIA-56392 (Svinjaricka Cuka)",3166,28);
      R_Date("KIA-56393 (Svinjaricka Cuka)",3190,40);
    };
  };

  Boundary("Southeastern_Europe_end");
};

Sequence("Caucasus")
{
  Boundary("Caucasus_start");

  Phase("Caucasus_sites")
  {
    Phase("Namcheduri")
    {
      R_Date("Poz-66777 (Namcheduri)",3150,35);
    };

    Phase("Gudabertka")
    {
      R_Date("Poz-66771 (Gudabertka)",2815,30);
    };

    Phase("Grakliani_Gora")
    {
      R_Date("Poz-51404 (Grakliani Gora)",2935,30);
    };

    Phase("Gegharot")
    {
      R_Date("Poz-63429 (Gegharot)",2995,30);
    };

    Phase("Tsaghkahovit")
    {
      R_Date("Poz-66779 (Tsaghkahovit)",2945,30);
    };

    Phase("Chishkho")
    {
      R_Date("Poz-82836 (Chishkho)",3000,30);
    };

    Phase("Chidgom")
    {
      R_Date("Poz-82835 (Chidgom)",2870,30);
    };

    Phase("Guamsky_Grot")
    {
      R_Date("GrA-65338 (Guamsky Grot)",2835,35);
    };
```

```
      Phase("Kabardinka_2")
      {
        R_Date("Poz-82838 (Kabardinka 2)",2950,30);
      };
    };

    Boundary("Caucasus_end");
  };

  Sequence("Anatolia")
  {
    Boundary("Anatolia_start");

    Phase("Anatolia_sites")
    {
      Phase("Sos_Hoyuk")
      {
        R_Date("Poz-66780 (Sos Hoyuk)",2945,35);
        R_Date("Poz-66781 (Sos Hoyuk)",2960,35);
      };

      Phase("Troy")
      {
        R_Date("FTMC-US70-1 (Troy)",2885,33);
        R_Date("FTMC-US70-2 (Troy)",2959,31);
      };
    };

    Boundary("Anatolia_end");
  };
};
```
